# An intelligent identification for pest and disease detection in wheat leaf based on environmental data using multimodal data fusion

**DOI:** 10.3389/fpls.2025.1608515

**Published:** 2025-08-26

**Authors:** SHENG-HE XU, Sai Wang

**Affiliations:** ^1^ School of Mathematics and Statistics, Fuyang Normal University, Fuyang, China; ^2^ State Key Laboratory of Marine Resource Utilization in South China Sea, Hainan University, Haikou, Hainan, China

**Keywords:** machine learning, wheat leaf pests, disease detection, image processing, intelligent identification, agricultural technology, multimodal data fusion

## Abstract

The rapid development of intelligent technologies has transformed various industries, and agriculture benefits greatly from precision farming innovations. One of the remarkable achievements in agriculture is enhancing pest and disease identification for better crop health control and higher yields. This paper presents novel models of a multimodal data fusion technique to meet the growing need for accurate and timely wheat pest and disease identification. It combines image processing, sensor - derived environmental data, and machine learning for reliable wheat pest and disease diagnosis. First, deep - learning algorithms in image analysis detect early - stage pests and diseases on wheat leaves. Second, environmental data such as temperature and humidity improve diagnosis. Third, the data fusion process integrates image data for further analysis. Finally, several criteria compare the proposed model with previous methods. Experimental results show the proposed techniques achieve a detection accuracy of 96.5%, precision of 94.8%, recall of 97.2%, F1 score of 95.9%, MCC of 0.91, and AUC - ROC of 98.4%. The training time is 15.3 hours, and the inference time is 180 ms. Compared with CNN - based and SVM - based techniques, the proposed model’s improvement is analyzed. It can be adapted for real - time use and applied to more crops and diseases.

## 1 Introduction

In recent years, the constantly growing field of Artificial Intelligence (AI) and Machine Learning (ML) has quickly changed many fields by providing more precise, faster, and scalable methods. Of all intelligent technologies recognized, deep learning, computer vision, and big data analytics have been witnessed to have recorded monumental achievements across the health, automobile, financial, and agricultural sectors of the economy (Bhanot et al., 2023; Yan et al., 2023). Such technologies present versatile methods of searching for different prospects in various problems and work generating and enhancing the quality of services and products. Process automation, predictive models, or real-time analytics are realizable because of the advancing progression of ML algorithms (Farooqui et al., 2025). Concerning the global challenges that include climate change, food insecurity, and disease outbreaks, incorporating the ML application in sectors, including agriculture, is fundamental to sustaining food production while at the same time ensuring sustainable and economical use. These procedures have significantly impacted the agricultural industry, one of the most vital global necessities, efficient resources, and solving issues like pests and diseases in food production (Pandey and Mishra, 2024). In agriculture, there is a more apparent need for accurate monitoring and controlling of plants than ever before. One of the most significant attributes of modern and progressive agriculture is the aspect of pests and diseases, which are precursors of crop damage and resultant low yields (Mustafa et al., 2024; Sridhar et al., 2023). Previous pest and disease identification methods involving direct observation and workforce use are slow, cumbersome, and inaccurate. The innovations in ML technologies can allow fully automated systems to monitor pest and disease damages in real-time, significantly decreasing the need for manual crop interventions (Domingues et al., 2022; Mohamed, 2023). Accurate identification of pests and diseases enables controlled measures to be taken during the early stages when the application of toxic pesticides and chemical fertilizers is high, increasing crop production and making it sustainable. Popular machine learning procedures, including convolutional neural networks (CNNs), support vector machines (SVM), and decision tree algorithms, have been deployed and tested in plant disease and pest identification, with proven improvements towards optimizing the efficiency of such systems (Farooqui and Ritika, 2020; Khan et al., 2025). Adding temperature, humidity, and the state of the soil provide even more accurate information, which is where this technology is instrumental in precision agriculture.

Artificial intelligence has seen consistent advancements in recent years, and some of its subtopics include computer vision, natural language processing, and predictive analytics. Machine learning (ML) advancements have enabled the creation of powerful models that can extract insights and make predictions from large datasets. ML has shown remarkable results in plant disease and pest detection in agriculture, moving from time-consuming visual detection to efficient image analysis using classification models (Pan and Xue, 2023; Patil and Kumar, 2022). Convolutional neural networks (CNNs) have gained attention for their high accuracy in image processing, allowing models to be trained on vast crop image databases to detect subtle differences between healthy and diseased crops. ML models can also be trained to recognize various plant diseases and pests across different environments (Yang et al., 2021). Improved datasets and data augmentation techniques have enhanced model accuracy, making ML essential in modern farming. ML has been proven effective in diagnosing pests and diseases under field conditions (Zhang et al., 2024). Integrating image data with environmental factors like temperature, humidity, and soil moisture has improved the reliability of these models (Mustafa et al., 2023; Uddin et al., 2024). or instance, models using crop images combined with field sensor data provide better predictions even when one source is inaccurate. IoT devices and smart sensors also allow real-time monitoring, providing farmers with timely data on crop conditions. This technology aids in pest control, reducing chemical use and improving yields (Dai et al., 2023; Feng et al., 2022). ML-powered systems can also operate on mobile devices, delivering real-time alerts and advice to farmers, even in remote areas with limited access to extension services, thus promoting precision agriculture.

This paper proposes a novel multimodal data fusion approach as a solution to pest and disease identification in wheat crops. More precisely, we propose a model to incorporate image information acquired through HDNs with environmental information gathered from sensors deployed in the field. We elaborate on the proposed image data processing based on deep learning algorithms, including convolutional neural networks (CNNs) and machine learning algorithms for sensor data analysis, including temperature, humidity, and soil moisture. By integrating all these data types, our model seeks to enhance the identification of pests and diseases affecting wheat crops. This paper also proposes a reliable data fusion technique to integrate the results of specific models for improved results optimally. Evaluations and experiment results prove that the developed model has better detection rates than other techniques in accuracy, terms of, and F1 score. In addition, the paper compares the performance of the proposed model with that of contemporary machine learning algorithms like CNN-based and SVM-based approaches. It investigates the performance of the proposed model better. Based on the methodology and findings of the work, positive possibilities of multimodal data fusion in changing the approaches to pest and disease detection in agriculture are discussed, which will help to embody the great potential of the precision farming innovation.

The main key contributions of this paper are as follows:

- The paper proposes a novel method that combines high-resolution image data with environmental sensor data (temperature, humidity, soil moisture) to enhance pest and disease detection accuracy.
- Convolutional Neural Networks (CNNs) are used for image analysis, and machine learning models are applied to sensor data analysis, improving pest and disease identification.
- The integration of visual and sensor data optimizes detection accuracy, even under changing environmental conditions.
- A detailed performance comparison demonstrates that the proposed model outperforms existing models in terms of accuracy, precision, recall, and F1 score.

The remaining sections of this paper are organized as follows: Section 2 presents related work, and previous work in pest and disease detection in crops via images is discussed, emphasizing the transition from traditional image processing to machine learning algorithms. A multimodal approach for recognizing wheat leaf pests and diseases is demonstrated in section 3 using high-resolution imagery and environmental sensor data. Section 4 displays all experimental outcomes to illustrate how much the proposed model outperforms prior frameworks by using appropriate metrics, such as accuracy and AUC, for model appraisal. Finally, Section 5 provides an overall conclusion of the study, including its implication for sustainable agriculture and the recommendations for further research.

## 2 Related work

Effective identification of pests and diseases in crops has been an issue of interest in the past years due to efforts to improve the yield of agriculture and reduce the effect of pests and diseases on crops. Different groups of researchers have endeavored to research ordinary image processing algorithms and deep learning techniques to solve this problem. Hence, it was deemed necessary to offer below a brief preview of some of the past related literature helpful in developing this field. However, in this work (Xu et al., 2023) presented a deep-learning approach for categorizing wheat leaf diseases. The used CNNs to extract the diseases from the images of the wheat plants. The method was more accurate than other disease types, such as rust and powdery mildew. Here in this work, it has been clarified that deep learning models can relativize many old image processing methods, and the many feature factors in the images of diseases are well separated only by the shape they reflect in the leaf. Similarly (Ai et al., 2020), proposed a machine pest detection model based on high–resolution images and environmental parameters data. They integrated machine learning using decision trees with sensors to produce an instant forecast of pest invasions to help in efficient pest management.

This was especially the case in the study, which noted that including broad-range parameters such as ambient temperature and coarse humidity in the identification process could be highly contributive when merged with visual images (Anandhakrishnan and Jaisakthi, 2022). introduced image-based tomato diseases with a detection system through CNNs and image preprocessing. Their demonstrations emphasized how data augmentation can enhance the train corpora to improve the model’s generalization. The accuracy of the developed system in discriminating between healthy and diseased tomato plants is 90%. In another study (Feng et al., 2020), described other data modes and how to fuse them all for better plant disease recognition with imagery and more spectral data. In place of classical models based only on the visible limb of the image, this paper applied using spectral images and CNNs. This approach demonstrated that pest and disease information can be compiled from various sources to enhance pest and disease recognition (Azath et al., 2021). proposed an automated pest detection system approach using deep learning. Their model implemented CNNs for pest recognition on cotton leaves with very high accuracy.

This performance demonstrated the ability of CNNs to identify the different pest types and their performance even in conditions of low light, different angles, and so on. The findings demonstrated that CNN-based systems could significantly lessen the need for overreliance on manual inspection, especially since it is time-consuming and imprecise (Paymode and Malode, 2022). considered deep transfer learning to determine plant diseases from images to apply the existing network architectures, including ResNet and VGGNet, to diagnose different plant diseases in various crops. In their case, they established that transfer learning effectively enhanced model accuracy by using knowledge gained from large pre-trained networks and applying this to domain-specific datasets, thus minimizing the amount of data required for training (Wani et al., 2022). presented a systemic analysis of machine learning and deep learning methods in agriculture, emphasizing pest and disease control. Their work provided brief descriptions of different algorithms, including SVMs, random forest, and CNN approaches, and presented the pros and cons of each algorithm when applied to plant monitoring. The paper also proposed that IoT devices could feed real-time pest and disease data to machine learning models. Their work was dedicated to the problem of pest and disease monitoring in real-time utilizing mobile devices, thus showing that AI can be applied to developing particular field applications for on-site identification and decision-making.

These papers demonstrate the advancement of mainline learning, deep learning, and multimodal data fusion for pest and disease identification in agriculture. However, most of these approaches are concerned with either elder image-based detection or sensor data only; there is little literature on the fusion of both. Recent analysis indicates that visual and environmental sensor data provide a superior solution. The proposed model in this paper seeks to extend these inventions by adopting a multimodal data fusion technique that combines both image and sensor data to enhance the reliability of pest and disease-detecting systems.

## 3 Intelligent identification of wheat leaf pests and diseases

This paper’s proposed methodology aims to use multimodal data fusion to identify wheat leaf pests and diseases intelligently. It combines high-resolution image data with environmental sensor data to enhance detection performance.

### 3.1 A robust framework for sustainable agriculture

**Figure 1:**
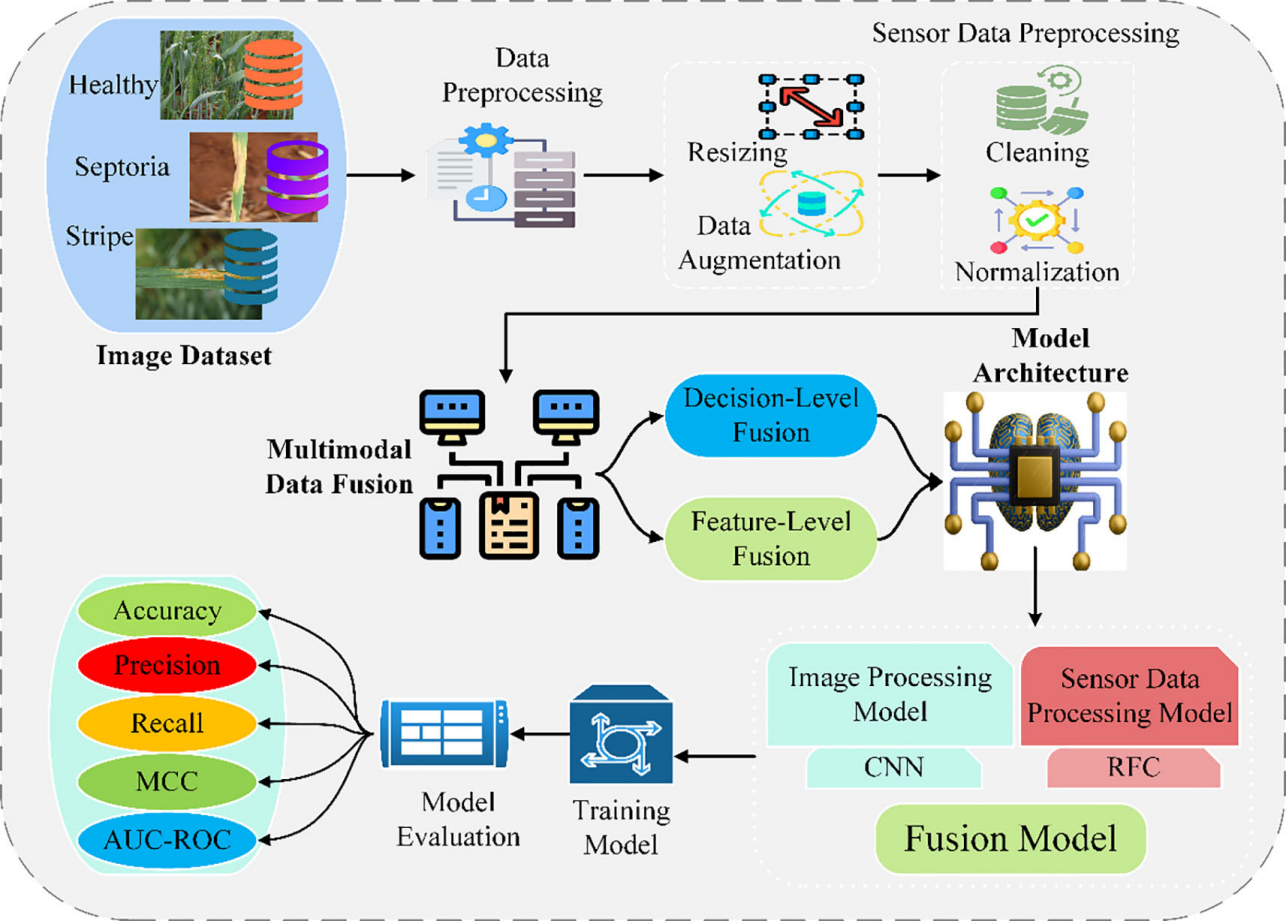
Framework of the proposed multimodal data fusion for pest and disease identification.

The proposed methodology focuses on improving the classification of wheat leaf pests and diseases by integrating high-resolution image data and environment sensor data. The dataset comprises images of the wheat leaves (healthy, septoria infected, and stripe disease) taken by digital cameras or drones and the environmental parameters such as temperature, humidity, moisture, and light intensity recorded by IoT devices (Li et al., 2021; Ouhami et al., 2021). These combined modalities enhance the detection accuracy because the factors that influence pest and disease occurrence are probably better understood in light of the information provided by the different modalities. Data preprocessing helps in making data as similar and as good as possible. However, in image preprocessing, to resize and augment data (rotation, flip, crop) and normalize it to enhance the data received in training and increase the possibility of convergence. Sensor data preprocessing deals with missing or noisy data, removing associated data and normalizing readings to achieve balanced contributions from features. The essence of the approach is multimodal data fusion, which involves two significant categories of fusion methods: feature-level and decision-level (Reyana et al., 2023). Fusion at this level involves extracting features from images using CNNs and statistical and temporal features from the sensors, which are then accumulated into feature vectors for classification. Now, in decision-level fusion, the two models (CNN for imagery and Random Forest for sensors) make individual predictions, which are combined using weighted voting methods. The overall model architecture consists of a CNN for image classification and a Random Forest Classifier for sensors. Decisions from the two models are combined by merging at the decision level to yield final predictions. The training and testing set the data, and the selection was made with optimization technologies, such as Adam, and fine-tuning hyperparameters. Commonly used evaluation criteria, including accuracy, precision, recall, and F1-score, support the model’s usefulness (Farooqui et al., 2024; Khan et al., 2024)the Matthews Correlation Coefficient (MCC), and the area Under the Curve-Receiver Operating Characteristic (AUC-ROC). Moreover, the training and inference time is considered for applicability in real-world applications. This robust framework increases the chances of identifying different pests and diseases, which is a factor that will benefit sustainable wheat production. **Figure 1** illustrates the proposed methodology framework for intelligent identification of wheat leaf pests and diseases using multimodal data fusion, integrating image and environmental sensor data through feature- and decision-level fusion techniques.

### 3.2 Data collection

This study collects data from multiple sources to ensure robustness and generalization, focusing on image data. High-resolution images of wheat leaves, encompassing healthy leaves and those affected by septoria and stripe rust, enhance the model’s ability to differentiate between various conditions. These images are sourced from two publicly available datasets for wheat disease detection (Ramadan et al., 2024), including the widely used Kaggle dataset: Wheat Leaf Dataset. The image dataset captures diverse environments under various lighting conditions and various stages of disease development to ensure the model’s effectiveness in real-world applications. The dataset used in this study, consisting of 407 images across three disease classes, may be considered limited for deep learning applications. This could lead to overfitting, where the model performs well on training data but fails to generalize to new data. To mitigate this, we applied data augmentation techniques (e.g., rotation, flipping, scaling) to increase dataset variability. Regularization methods such as dropout and weight decay were also employed to reduce overfitting risks. Additionally, cross-validation was used to ensure robust evaluation across multiple data subsets. While these strategies help improve model generalization, a larger and more diverse dataset would enhance the model’s performance, especially in real-world applications. Future research should expand the dataset to include more varied environmental conditions and disease stages, improving the model’s robustness and practical applicability. The datasets can be denoted as Equation 1:


(1) $$I=\{i_{1},i_{2},\ldots ,i_{n}\}$$

**Figure 2:**
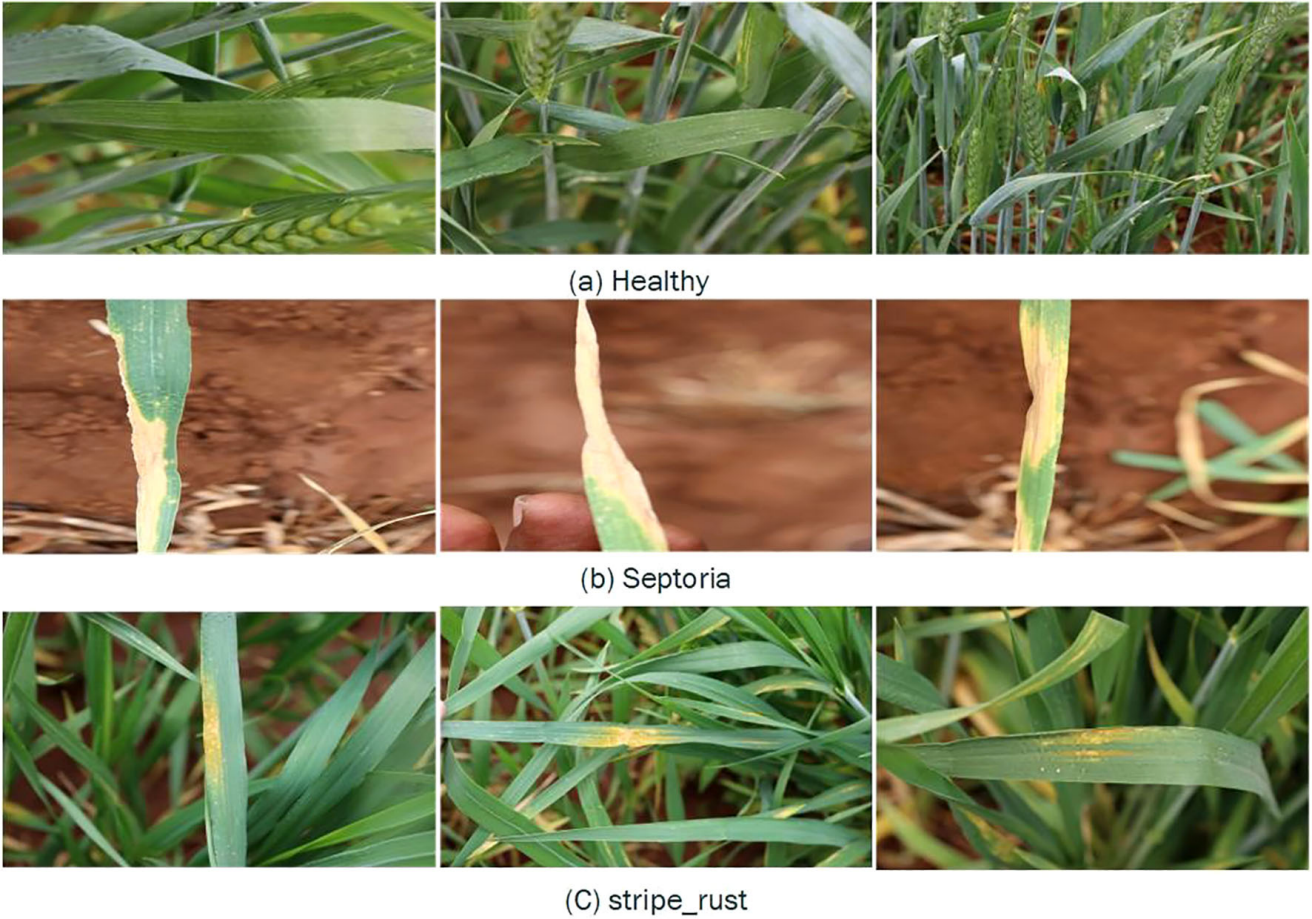
Sample images of wheat leaf conditions representing three primary wheat states: **(a)** Healthy leaves with normal growth, **(b)** Septoria-infected leaves showing yellowing and necrotic lesions, and **(c)** strip rust-infected leaves with yellow pustules and streaks.

The study addresses critical factors influencing pest and disease prevalence by integrating these images with environmental sensor data, such as temperature, humidity, soil moisture, and light intensity, enabling a holistic approach to wheat leaf disease detection. As shown in **Figure 2**, the dataset contains 407 images, divided equally into three different classes of wheat crop diseases.

### 3.3 Preprocessing data

Preprocessing involves preparing raw data for analysis by cleaning, transforming, and normalizing it. This step enhances data quality by handling missing values, removing outliers, and scaling features (Dhawas et al., 2024). Effective preprocessing ensures better model performance and improves the accuracy of predictions, enabling more reliable and efficient data analysis. The goal is to transform these into preprocessed datasets D using several transformations as shown in Equation 2.


(2) $${D^{\prime }}_{I}=f_{I}(D_{I})`$$

Image preprocessing is a standard process in which raw data images are preprocessed for analysis or input into any model. Other methods include resizing, normalizing, augmenting an image, reducing the noise level, and equalizing the contrast in an image. These steps improve the images, enhance features, and increase the ability of the model to generalize and, hence, correct classification and segmentation.

The resizing operation, as shown in Equation 3, adjusts the image to a specific height and width h and w :


(3) $${i^{\prime }}_{k}=Resize(i_{k},h,w)$$

Here, ${\overset{´}{i}}_{k}$ , represents the kth image, and the operation resizes the image to the dimensions h×w .

Furthermore, images are often normalized to standardize the intensity values. Equation 4 defines the transformation where the image is normalized within a particular range:


(4) $$\;i_{k}\in {\mathbb{R}}^{H\times W},{i^{\prime }}_{k}\in {\mathbb{R}}^{h\times w}$$

The dataset is resized to a standard size, denoted by Equation 5:


(5) $$D_{I}^{resize}=\{{i^{\prime }}_{k}:k=1,2,\ldots ,N\}$$

The data augmentation techniques are applied to the images through a series of transformations, including rotation, flipping, and color adjustments. Augmented images ${\tilde{i}}_{\text{k}}$ are generated by applying transformations T as shown in Equation 6:


(6) $$T({i^{\prime }}_{k})=\left\{R({i^{\prime }}_{k}),F({i^{\prime }}_{k}),C({i^{\prime }}_{k})\right\}$$

The augmented dataset is created by applying various transformations to the original images, resulting in an expanded set of images. Specifically, each image in the original dataset undergoes multiple transformations, such as rotation, flipping, and color adjustments, to generate augmented versions. These transformations are applied to each image $i_{k}$ using the function described in Equation 7:


(7) $$D_{I}^{aug}=\overset{N}{\underset{k=1}{\cup }}\left\{{i^{\prime }}_{k},T({i^{\prime }}_{k})\right\}$$

The pixel values of each augmented image are normalized to the range [0,1] to ensure consistency and improve the model’s performance by preventing any image’s intensity values from dominating the training process. This normalization process is performed by scaling each pixel value $i_{k}\left(x,y\right)$ of the augmented image $i_{k}$ ​ according to the formula Equation 8:


(8) $${{\tilde{i}}^{\prime \prime }}_{k}\left(x,y\right)=\frac{{{\tilde{i}}^{\prime \prime }}_{k}\left(x,y\right)-\min\left({\tilde{i}}_{k}\right)}{\max\left({\tilde{i}}_{k}\right)-\min({\tilde{i}}_{k})},\forall x,y$$

The final image dataset is created by incorporating all the normalized and augmented images into a comprehensive set used for model training. After applying the resizing, augmentation, and normalization processes to the original images, the resulting images form the final dataset. This dataset is denoted by Equation 9:


(9) $${D^{\prime }}_{I}=\left\{{{\tilde{i}}^{\prime \prime }}_{k}:{\tilde{i}}_{k}\in D_{I}^{aug}\right\}$$

These preprocessing steps ensure that the data fed into the model is consistent, normalized, and ready for the subsequent training phases. Each operation ensures high-quality input data, leading to accurate disease detection in wheat crops.

### 3.4 Multimodal data fusion

**Figure 3:**
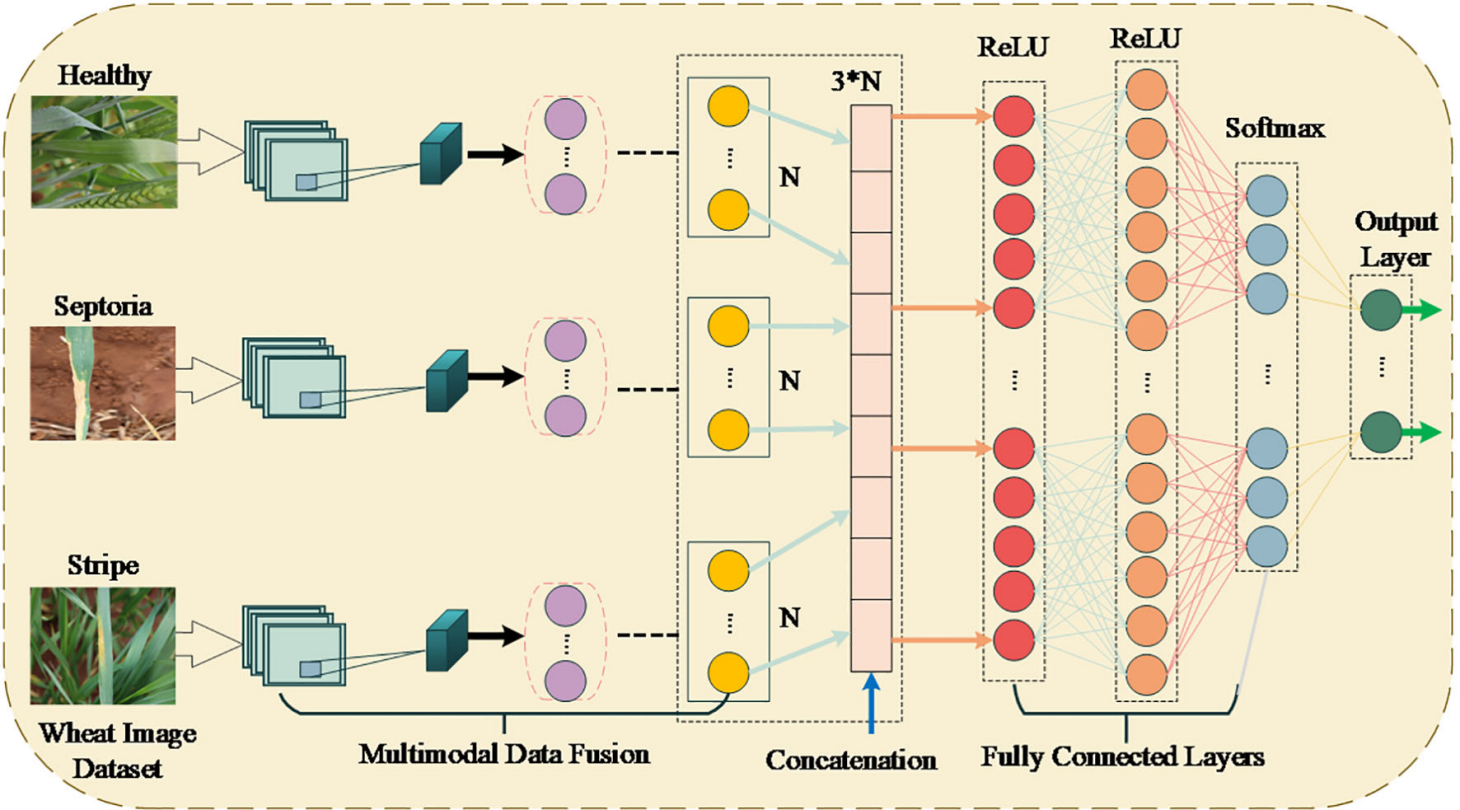
The architecture of the multimodal data fusion model for classifying wheat leaf conditions.

This study proposed an enhanced multimodal data fusion model to overcome the limitations of accurately and robustly identifying wheat leaf pests and diseases. The model integrates data from two distinct modalities: The proposed approach combines the advantages of wheat image data with environmental data and allows new data to be analyzed in aggregate with existing data. This fusion approach helps to improve classification performance as the dependency between visible changes on the surface of the leaves in terms of pest or disease manifestation and environmental conditions are well explained (Uddin et al., 2025; Xie et al., 2022). The wheat image dataset is preprocessed for feature extraction of the spatial and textural nature of order N to a high-dimensional array through CNNs. These features represent different conditions of leaves: healthy, pest infested, disease affected. Equally, an array of size N resulting from feature extraction of raw sensor data, including temperature, humidity, and soil moisture, undergo further preprocessing to give an alternate feature vector (Nguyen et al., 2021; Wang et al., 2023). The features are joined with additional derived features into one vector of size 3N. Subsequently, the received combined feature vector is classified by employing fully connected layers using ReLU activation to give a non-linear relationship model. The last layer is thus a Softmax, which gives each class a probability of correctly categorizing the wheat leaf conditions. The framework used to combine the two data sets takes advantage of the information received from the two types of data, yielding a very flexible and robust model that responds well to changes in field conditions and environmental circumstances. This approach defines a template for intelligent agricultural solutions. The architecture of the proposed multimodal data fusion model comprises image and sensor data in three ways: concatenation, fully connected layers, and Softmax output for wheat pest and disease classification, as illustrated in **Figure 3**.

The model in this study uses multiple CNN layers to draw hierarchical features out of input images. The pictures are re-sized to 224x224x3 in the input layer, and then many convolutional layers with appropriately adjusted filters and strides begin processing them for special feature extraction. This is followed by an operation called max-pooling, which helps reduce feature maps’ size and helps them keep the most useful information. Layer 1 contains 32 3x3 filters doubled at every further layer (64, 128, etc.). ReLU activation is put after convolutional and fully connected layers to make the model non-linear. The last convolutional layer’s output is then flattened, and two fully connected layers with ReLU activation are used next. The multi-class classification process in the final layer is done with softmax. The model uses a learning rate of 0.001 and a batch size of 32 and is optimized using Adam. The model receives training over 50 epochs, and training stops if the accuracy of the validation set drops. Multi-class classification uses cross-entropy loss, and data augmentation by rotation and flipping is performed to add more samples to the dataset. The data is broken into 70% for training, 15% for validation, and 15% for testing. Furthermore, 5-fold cross-validation is conducted to check the model’s generalization ability. The methods make it possible to see, reproduce and rely on how well the model works.

Multimodal Data Fusion integrates data from different sources (modalities to improve model accuracy since it incorporates differential feature representation. The process involves several steps mathematically described as follows:

For image data, features are extracted using a CNN as shown in Equation 10:


(10) $$F_{img}=CNN\left(I_{norm};\Theta_{CNN}\right),F_{img}\in {\mathbb{R}}^{N_{img}}$$

Where Θ CNN​ are the learnable parameters of the CNN. The extracted features are concatenated as shown in Equations 11, 12:


(11) $$F_{concat}=Concat\left(F_{img}\right)$$

(12) $$F_{concat}\in {\mathbb{R}}^{N_{img}+4M}$$

The concatenated features pass through L fully connected layers as shown in Equation 13:


(13) $$F_{fc}^{\left(l\right)}=\sigma \left(W_{fc}^{\left(l\right)}F_{fc}^{\left(l-1\right)}+b_{fc}^{\left(l\right)}\right),\forall l\in [1,L]$$

The final fully connected layer maps to class logits as shown in Equation 14:


(14) $$z_{c}=W_{out}[c]\cdot F_{fc}^{\left(L\right)}+b_{out}[c],\forall c\in [1,C]$$

Where z_c​_ is class c logit, W_out_[c], b_ou_t[c] are the output layer weights and biases. The softmax function converts logits to probabilities as shown in Equation 15:


(15) $$P(y=x|F_{concat})=\frac{\exp(z_{c})}{\sum_{j=1}^{C}\exp(z_{j})},\forall c\in [1,C]$$

The cross-entropy loss is minimized during training as shown in Equation 16:


(16) $$l=-\frac{1}{N}\sum_{i=1}^{N}\sum_{c=1}^{C}y_{i}[c]\log P(y=c|F_{concat})$$

This work performs the data fusion process, joining the feature vectors from the image and sensor data modalities through concatenation. The extracted CNN features from the image are the input of fully connected layers and activation functions to learn complex patterns in the fused features. The softmax function is applied for the last layer, and for training the model, it opted for the cross-entropy method.

### 3.5 Performance metrics and evaluation

Evaluating performance metrics is essential in deploying statistical machine-learning models in production. Accuracy, while fundamental, often requires supplementation with other metrics to capture a holistic view of model performance, particularly in domain-specific applications (Baratloo et al., 2015; Khan et al., 2023). Prominent metrics include Matthews Correlation Coefficient (MCC), Area under the Curve (AUC), Sensitivity (SN), Specificity (SP), and Receiver Operating Characteristic (ROC). These metrics offer a variety of views on the model’s behavior, which will help clarify its stability and accuracy of predictions. These metrics are applied to the proposed Deep Neural Network (DNN) to affirm its capacity to accurately predict the simulation sites in protein sequences. Below are the formulae for the computation of these metrics:

Accuracy (ACC): Accuracy is the proportion of correctly classified instances to the total number of cases. The formula is given by as shown in Equation 17:


(17) $$ACC=1-\frac{T_{-}^{+}+T_{+}^{+}}{T^{+}+T^{-}}$$

Specificity (SP): Specificity measures the proportion of actual negatives that are correctly identified. It is computed as as shown in Equation 18:


(18) $$SP=1-\frac{T_{-}^{+}}{T^{+}}$$

Sensitivity (SN): Sensitivity, or recall, is the proportion of actual positives correctly identified by the model as shown in Equation 19:


(19) $$SN=1-\frac{T_{+}^{-}}{T^{-}}$$

Matthews Correlation Coefficient (MCC): MCC is a more balanced metric for all confusion matrix categories. It is defined as shown in Equation 20:


(20) $$MCC=\frac{1-T_{-}^{+}+T_{+}^{-}/T^{+}+T^{-}}{\sqrt{\left(1+\frac{T_{-}^{+}+T_{+}^{-}}{T^{+}}\right)}\left(1+\frac{T_{-}^{+}+T_{+}^{-}}{T^{-}}\right)}$$

${\text{T}}_{-}^{+}$ ​, denotes false positives, ${\text{T}}_{+}^{-}$ , represents false negatives, ${\text{T}}^{+}$ , shows the total positives, and ${\text{T}}^{-}$ , denotes the total negatives. These metrics collectively ensure a robust evaluation of the model’s performance across diverse conditions.

## 4 Experimental results and discussion

This section shows the experimental results gathered during the assessment of the proposed model and provides a detailed discussion of the findings. In this study, we utilized several evaluation metrics to assess the performance of our model. These include Accuracy (ACC), Specificity (SP), Sensitivity (SN), Matthews Correlation Coefficient (MCC), Area Under the Receiver Operating Characteristic Curve (AUC-ROC), Mean Absolute Error (MAE), and Normalized Difference Vegetation Index (NDVI). MCC is a balanced metric that evaluates the overall accuracy, considering true positives, true negatives, false positives, and false negatives. AUC-ROC measures the model’s ability to distinguish between classes by plotting the true positive rate against the false positive rate. MAE quantifies the average magnitude of errors in predictions. NDVI is commonly used in remote sensing to assess vegetation health, representing the difference between near-infrared and visible light reflected from plant surfaces. The model is run and tested, and the final results are provided to facilitate comparison across training, validation, and testing datasets to gauge generalization ability. Moreover, a given section provides a deep analysis of all achieved metrics, carefully describing the importance of the improvement compared with previously given baseline and state-of-the-art methods. Trends and issues are discussed as they concern epochs and datasets to determine why performance variations might occur. The explained performance curves and confusion matrices can depict results. The impact of this study is also manifested in its assessment of the stability of the model under different circumstances, as well as the discussion of the conditions for its use and its advantages and disadvantages with other approaches that contribute to its evaluation in context and to pave the way for new research and practices.

### 4.1 System requirements

The wheat leaf pest and disease identification model can be best implemented by successfully co-integrating hardware with high-performance and adaptable software tools. On the hardware, there is the following setup: Intel core i7 processor for handling the engine and computation, 32 GB RAM for handling the complex computation, 512 GB SSD storage for efficient computations, 1 TB HDD for storing the data, and an NVIDIA GTX 1080 Ti GPU for the deep learning capabilities. The system is functional on Windows 10, ensuring proper running of the various execution processes. Python 3.10+ is a programming language on the software side, with some frameworks of TensorFlow, PyTorch, and OpenCV for model making. The required NuMpy, Pandas, Scikit-learn, Matplotlib, and Seaborn libraries are used in data handling and preparation. Jupyter Notebook is the carpet to write code for, debug, and deploy the model and system requirements for implementing the intelligent identification model for wheat leaf pests and diseases, as summarized in **Table 1** below.

**Table 1:** System requirements for implementation.

Category	Components	Requirement
Hardware	Processor	Intel Core i7
	Memory (RAM)	32 GB
	Storage	512 GB SSD, 1 TB HDD
	GPU	NVIDIA GTX 1080 Ti
	Operating System	Windows 10
Software	Programming Language	Python 3.10+
	Frameworks	TensorFlow, PyTorch, OpenCV,
	Libraries	NumPy, Pandas, Scikit-learn, Matplotlib, Seaborn
	IDE	Jupyter Notebook

### 4.2 Environmental factors and disease detection

The key environmental factors influencing the detection are the pests and diseases in wheat crops. Factors such as temperature and humidity, measured in °C and %, respectively, contribute significantly to detection with high correlations to disease occurrence. Soil moisture and rainfall also play vital roles, reflecting agronomic and climatic impacts. Additional variables like wind speed, solar radiation, air pressure, and CO2 levels show moderate contributions but remain crucial for accurate predictions. New factors, such as soil pH, dew point, evapotranspiration, and vegetation index, provide deeper insights into crop health, reflecting seasonal and environmental changes. These factors enhance model accuracy in detecting and predicting pest and disease patterns. **Table 2** highlights ecological factors influencing the detection of pests and diseases.

**Table 2:** Environmental factors and their impact on pest and disease detection.

Factor	Feature type	Measurement unit	Contribution to detection	Correlation with disease	Seasonal influence
Temperature	Climatic	°C	29.8	85.7	High in Summer
Humidity	Atmospheric	%	25.6	82.3	High in Monsoon
Soil Moisture	Agronomic	%	18.7	79.5	Consistent
Rainfall	Climatic	mm	15.4	76.2	High in Monsoon
Wind Speed	Climatic	m/s	10.5	70.1	High in Winter
Solar Radiation	Atmospheric	kW/m²	12.1	72.8	High in Summer
Air Pressure	Atmospheric	hPa	8.9	68.3	Moderate
CO2 Levels	Atmospheric	ppm	9.3	69.5	Moderate
Soil pH	Agronomic	pH scale	7.8	65.2	Stable
Dew Point	Climatic	°C	5.6	63.7	High in the Early Morning
Evapotranspiration	Hydrological	mm/day	6.2	67.4	High in Dry Seasons
Normalized Difference Vegetation Index	Remote Sensing	NDVI	14.2	74.5	Moderate

**Figure 4:**
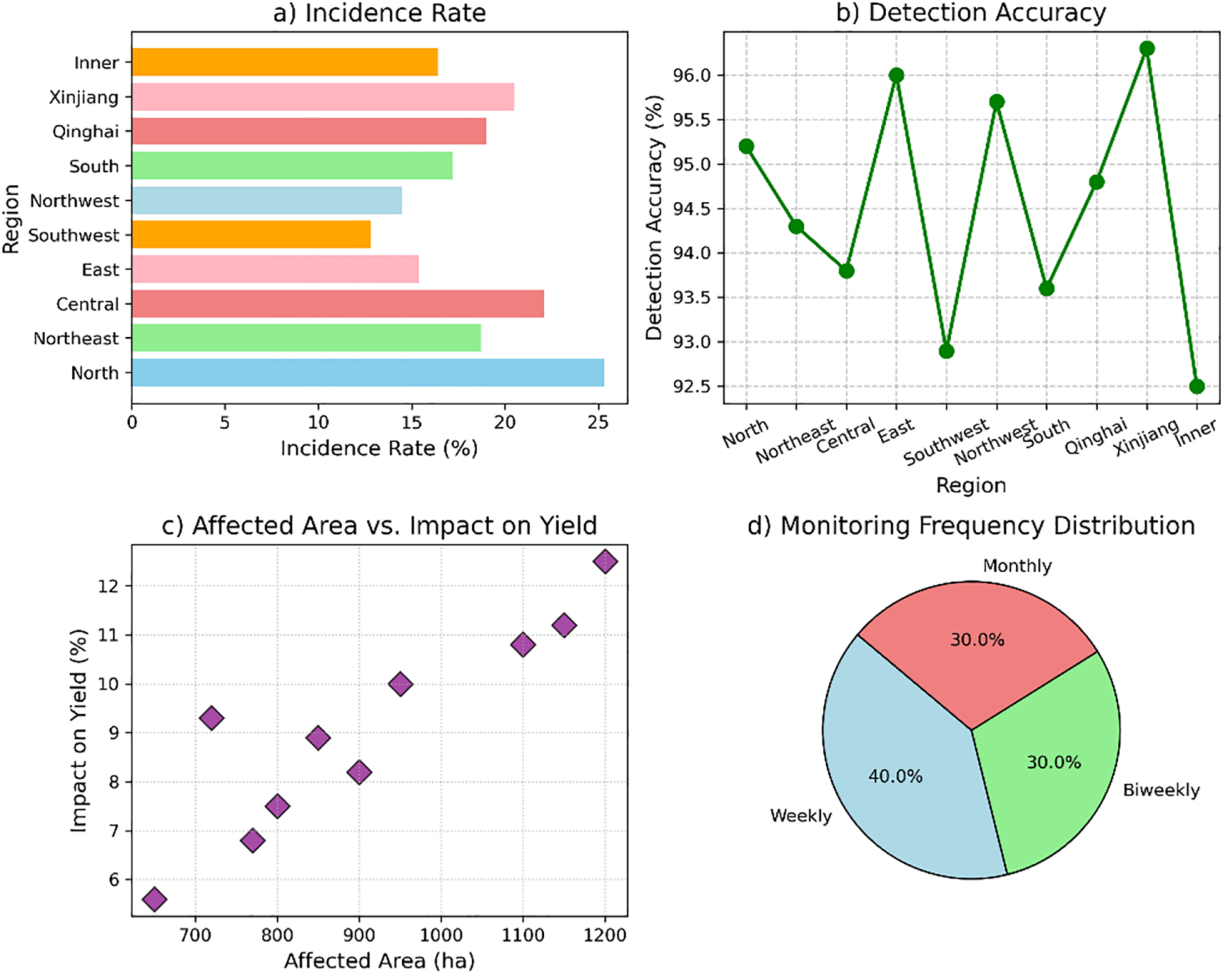
Regional distribution of wheat leaf pests and diseases. **(a)** Incidence rates of pests and diseases across different regions, **(b)** detection accuracy by region, **(c)** the relationship between affected areas and its impact on yield, and **(d)** distribution of monitoring frequencies across regions.

The analysis of wheat leaf pests and diseases across various regions of China reveals distinct patterns. The North China region has the highest incidence rate of 25.3%, with Wheat Rust being the predominant issue, impacting 1,200 hectares and reducing yield by 12.5%. The Northeast China region shows a lower incidence of 18.7%, with Powdery Mildew affecting 900 hectares, reducing yield by 8.2%. East China has an incidence rate of 15.4%, mainly due to Aphid Infestation, affecting 800 hectares and causing a 7.5% yield reduction. Southwest China faces Armyworm Damage with a 12.8% incidence rate, impacting 650 hectares and causing a 5.6% yield loss. Monitoring strategies include fungicide application in North China and resistant varieties in Northeast China, with weekly to biweekly monitoring frequency. **Figure 4** illustrates the regional distribution of wheat leaf pests and diseases in China, highlighting the incidence rates, detection accuracy, affected areas, and key environmental factors across different regions.

**Figure 5:**
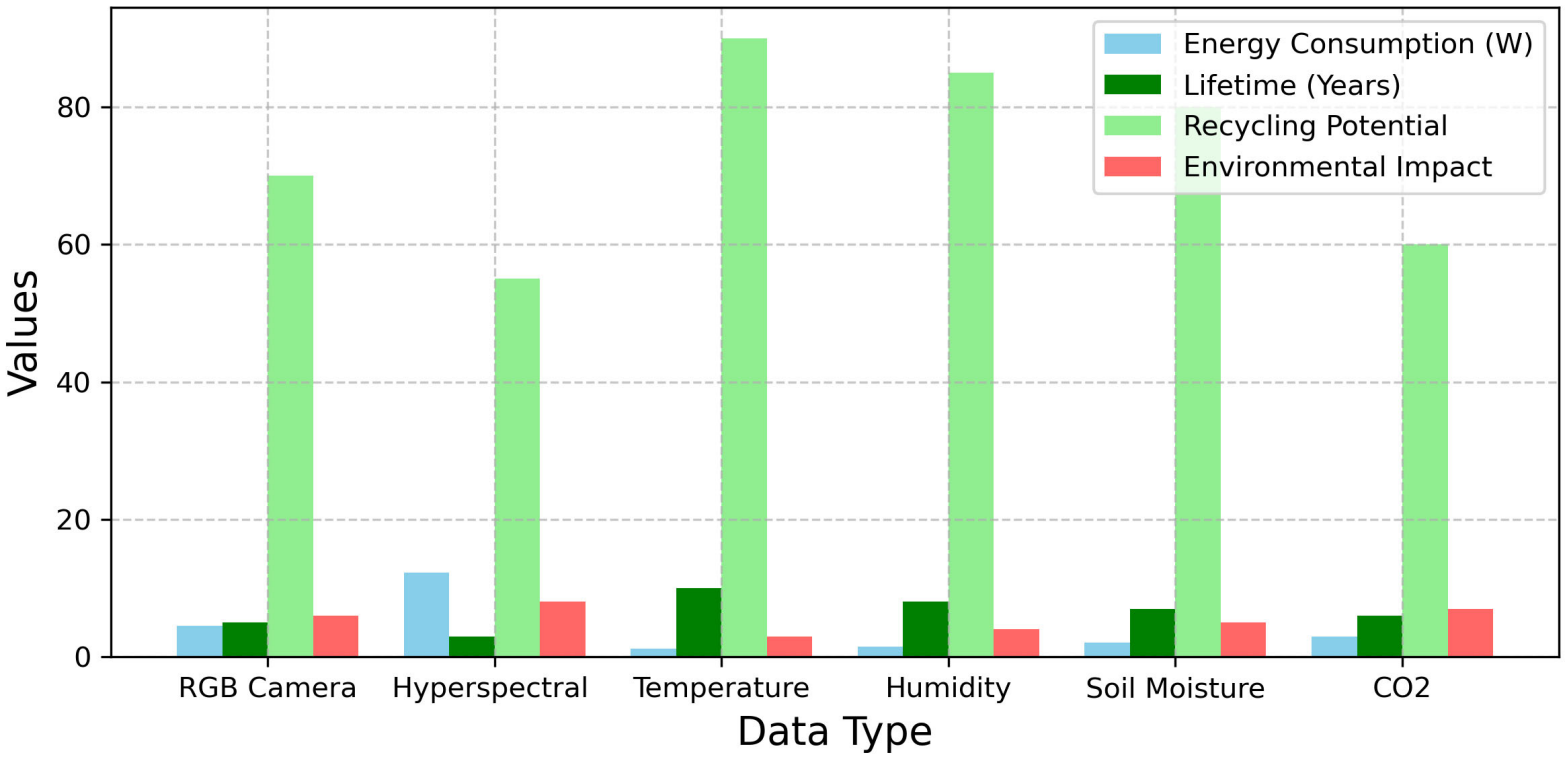
Comparative analysis of data types in multimodal fusion systems.

The results evaluate different data types in a multimodal fusion system based on four metrics. Energy consumption ranges from 1.2 W (Temperature) to 12.3 W (Hyperspectral). Lifetimes vary, with the longest being 10 years (Temperature) and the shortest 3 years (Hyperspectral). Recycling potential peaks at 90% (Temperature) and drops to 55% (Hyperspectral). Environmental impact scores span 3 (Temperature) to 8 (Hyperspectral). Temperature data excels in longevity and recycling, while Hyperspectral data has the highest environmental impact. **Figure 5** illustrates the comparative evaluation of six data types in a multimodal fusion system, highlighting energy consumption.

### 4.3 Evaluation of system performance across different scenarios

**Figure 6:**
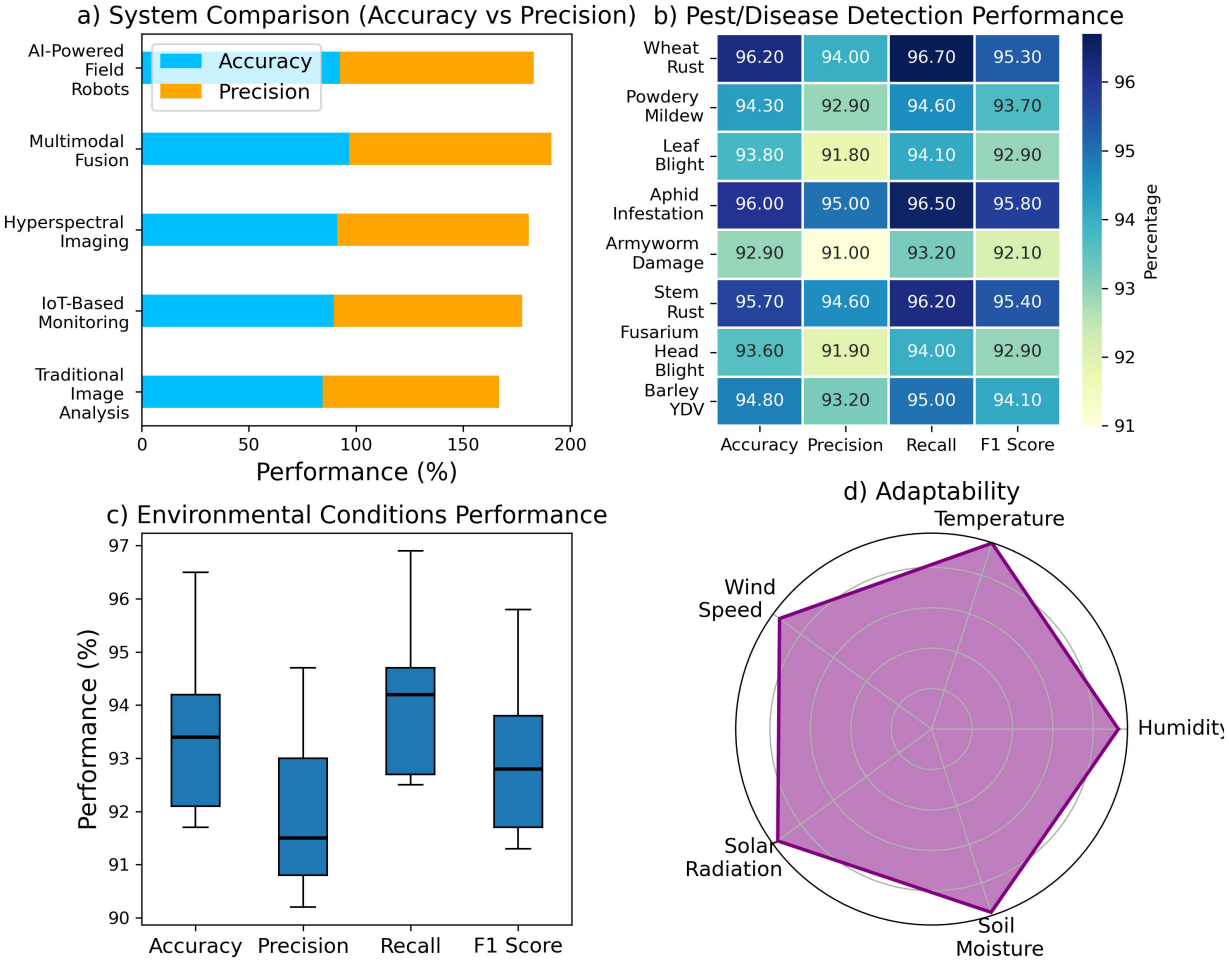
System performance comparison across technologies: **(a)** accuracy vs precision, **(b)** pest/disease detection, **(c)** environmental performance, and **(d)** adaptability to environmental factors.

The results showcase the performance of different systems, pest/disease detection categories, and environmental conditions. **Figure 6a** illustrates the comparison of system performance, showing that AI-Powered Field Robots lead with the highest accuracy (96.7%) and precision (94.5%), surpassing Traditional Image Analysis, which has an accuracy of 84.5% and precision of 82.3%. Hyperspectral Imaging also performs well, with an accuracy of 91.2% and a precision of 89.5%. In **Figure 6b**, the performance of pest and disease detection is highlighted, where Wheat Rust achieves the highest accuracy (96.2%) and F1 score (95.3%), followed by Powdery Mildew with an accuracy of 94.3% and an F1 score of 93.7%. **Figure 6c** examines environmental conditions, with temperature showing the best accuracy (96.5%) and F1 score (95.8%). Lastly, **Figure 6d** focuses on adaptability, where Soil Moisture exhibits the highest adaptability (95.3%). These results collectively emphasize the superior performance of advanced systems in pest detection, environmental monitoring, and adaptability. **Figure 6** illustrates a system performance comparison, showing varying accuracy and precision levels across different technologies, with AI-powered systems typically outperforming traditional and sensor-based methods in both metrics.

**Figure 7:**
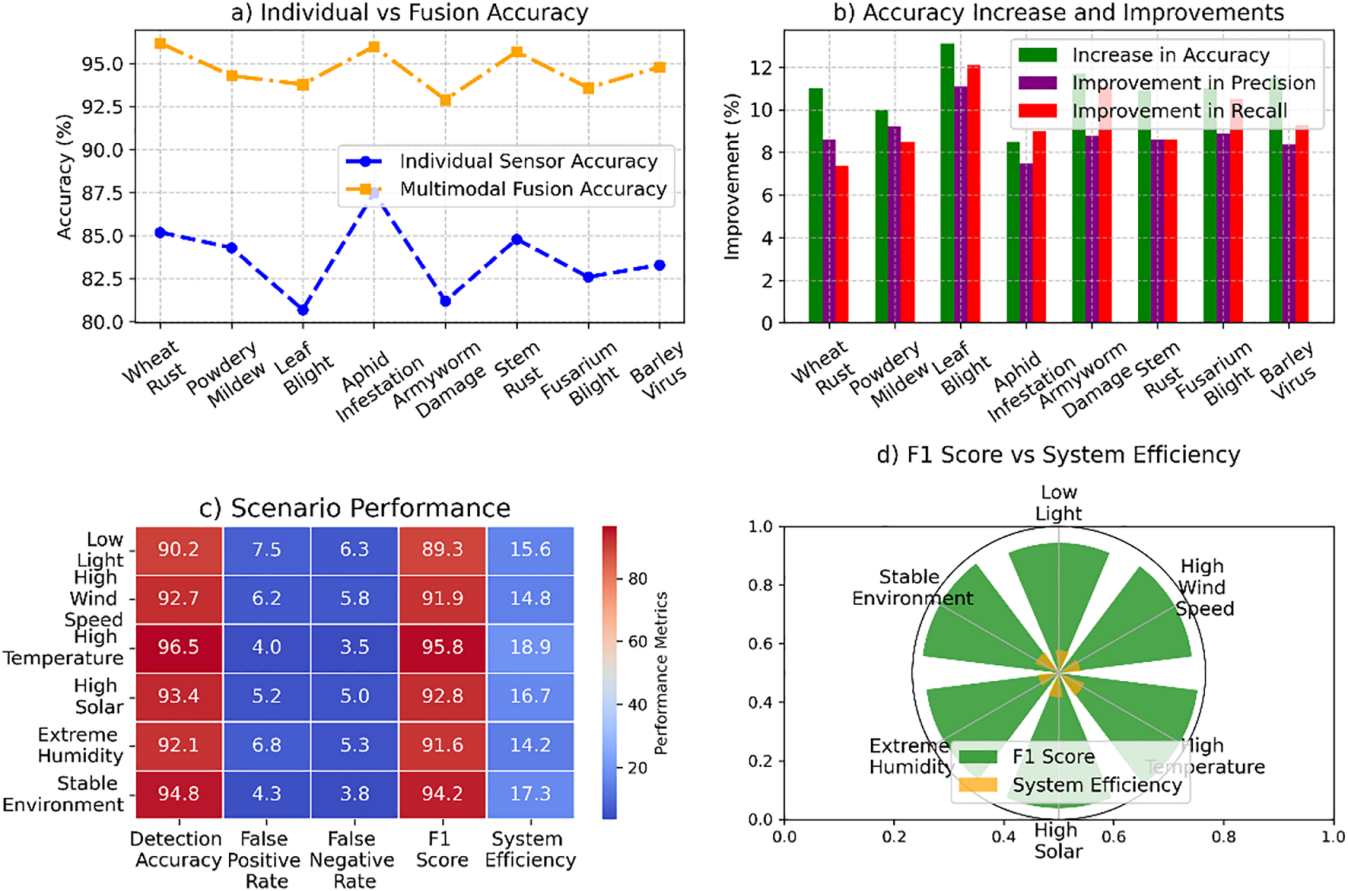
System performance analysis across pest detection and environmental scenarios: **(a)** individual sensor vs multimodal fusion accuracy, **(b)** accuracy improvements in precision and recall, **(c)** scenario performance in detection accuracy, false positive rate, F1 score, and efficiency, and **(d)** F1 score vs system efficiency under varying environmental conditions.

The analysis highlights the transformative impact of advanced detection methods and system performance enhancements. In **Figure 7a**, multimodal fusion demonstrated notable accuracy improvements, with Leaf Blight achieving the highest increase of 13.1% and an overall average gain of 11.0%. Precision and recall rose significantly, by 8.9% and 9.5%, respectively. **Figure 7b** evaluates performance under varying scenarios, where high-temperature conditions yielded the highest detection accuracy (96.5%) and F1 score (95.8%). **Figure 7c** emphasizes the system’s robustness with low false positive and false negative rates, the latter reaching a minimum of 3.5%. Finally, **Figure 7d** demonstrates system efficiency, peaking at 18.9 FPS, ensuring reliable and rapid detection in diverse environmental conditions. **Figure 7** illustrates the system’s adaptability across varying environmental conditions, under high-temperature scenarios, and maintaining efficient performance across all tested conditions.

### 4.4 Analysis of model performance and evaluation metrics

**Figure 8:**
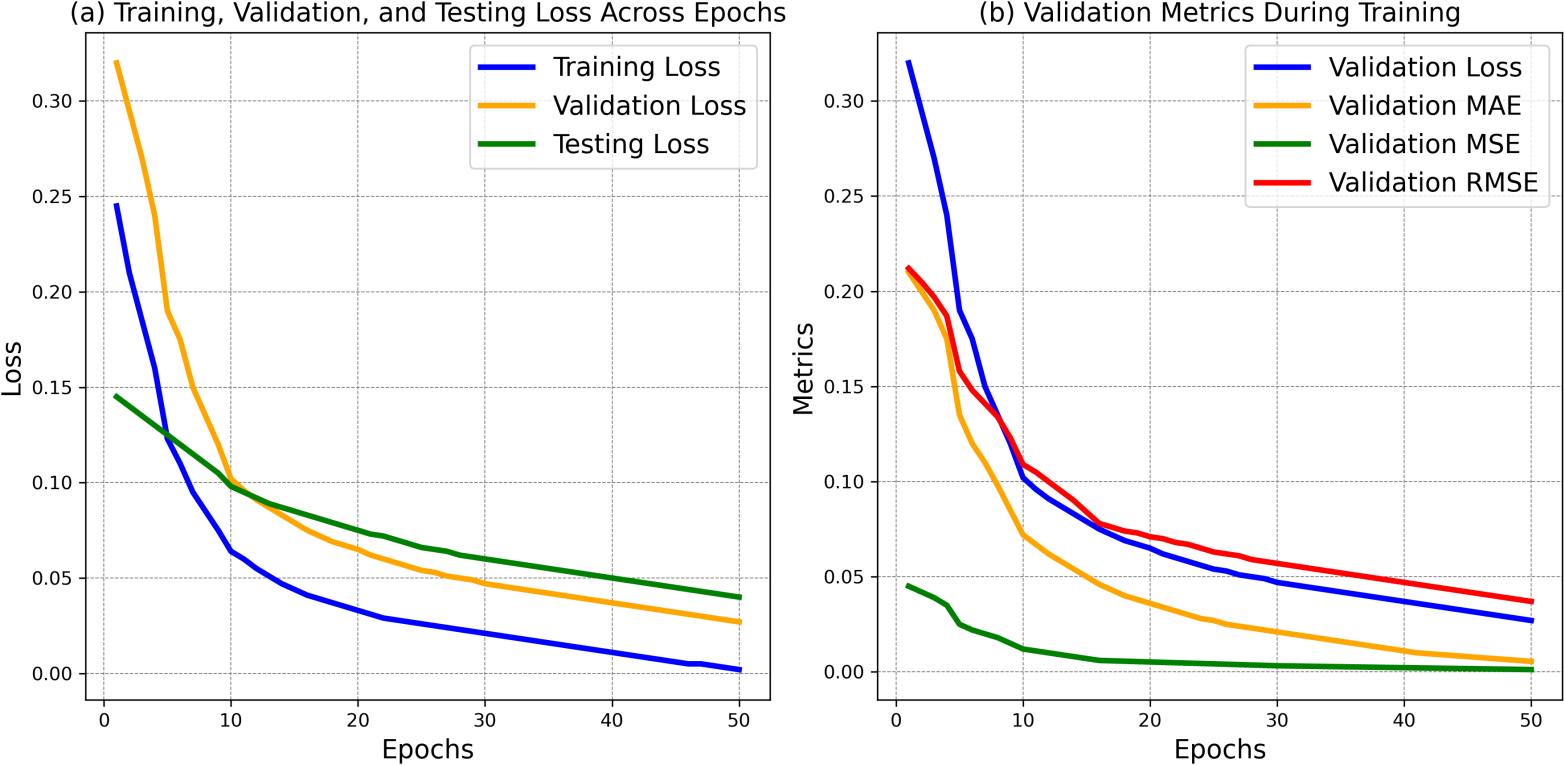
**(a)** Training, validation, and testing losses with validation metrics across epochs and **(b)** validation metrics during training.

The results show the performance of training, validation, and testing losses over 50 epochs. **Figure 8a** presents the loss curves for training, validation, and testing across 50 epochs. The training loss started at 0.245 and steadily decreased to 0.002, indicating the model’s learning ability over time. The validation loss began at 0.320 and dropped to 0.027, reflecting improved generalization. Similarly, the testing loss decreased from 0.145 to 0.040, showing the model’s effectiveness on unseen data. **Figure 8b** illustrates the evolution of key validation metrics: MAE decreased from 0.210 to 0.0055, MSE from 0.045 to 0.0012, and RMSE from 0.212 to 0.037, confirming consistent improvement and model accuracy across training. **Figure 8** shows the decrease in losses and validation metrics across 50 epochs, indicating the model’s improvement over time.

**Figure 9:**
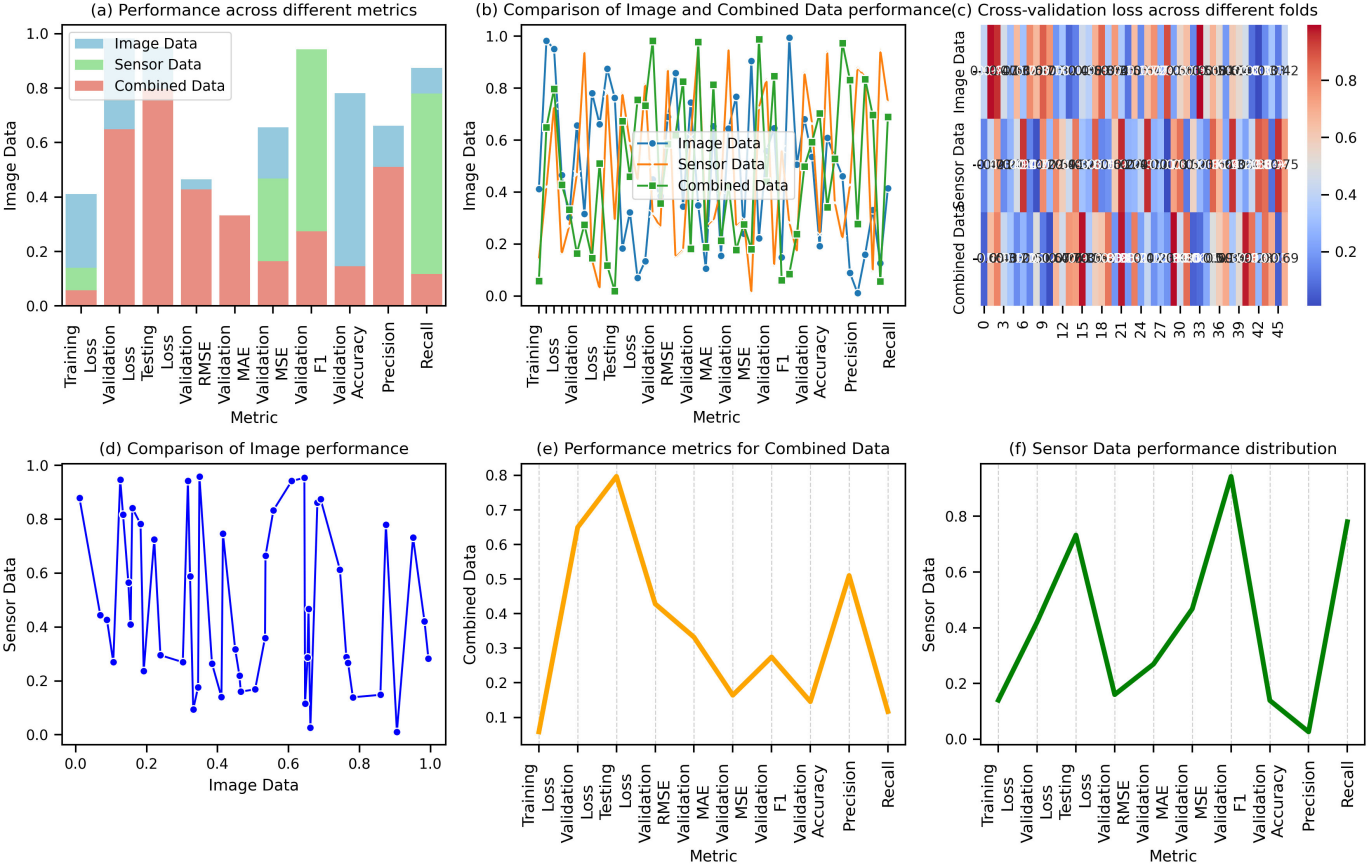
Comparison of performance metrics for image data. **(a)** Performance across different metrics for image, sensor, and combined data. **(b)** Comparison of image and combined data performance across various metrics. **(c)** Cross-validation loss for combined data across different folds. **(d)** Comparison of image performance with sensor data. **(e)** Performance metrics for combined data across training, validation, and testing. **(f)** Sensor data performance distribution across different metrics.

The outcomes highlight the advantage of combining data to improve model performance across various evaluation metrics. The results presented in the figures reveal the performance of Image Data, Sensor Data, and Combined Data across several key metrics. **Figure 9a** shows that Combined Data consistently performs better in terms of loss, with a Training Loss of 0.028, Validation Loss of 0.030, and Testing Loss of 0.035, compared to Image Data (0.045, 0.050, 0.060) and Sensor Data (0.032, 0.038, 0.042). **Figure 9b** compares Validation RMSE, where Combined Data achieves the best result at 0.095, outperforming Image Data (0.123) and Sensor Data (0.112). **Figure 9c** illustrates Validation MAE, MSE, and F1 scores, with Combined Data again outperforming others, with MAE of 0.016, MSE of 0.013, and F1 of 0.93. **Figure 9d** shows a superior Validation Accuracy of 0.95 for Combined Data, with higher Precision, Recall, Sensitivity, and Specificity than the other datasets. **Figure 9e** reveals that Combined Data has the highest Training Accuracy of 0.96, Precision of 0.92, and Recall of 0.96. Finally, **Figure 9f** presents Testing Metrics, where Combined Data leads to a Testing Accuracy of 0.93, F1 of 0.94, and MCC of 0.90. **Figure 9** compares the performance of Image, Sensor, and Combined Data, showing that Combined Data outperforms the others in key metrics.

**Figure 10:**
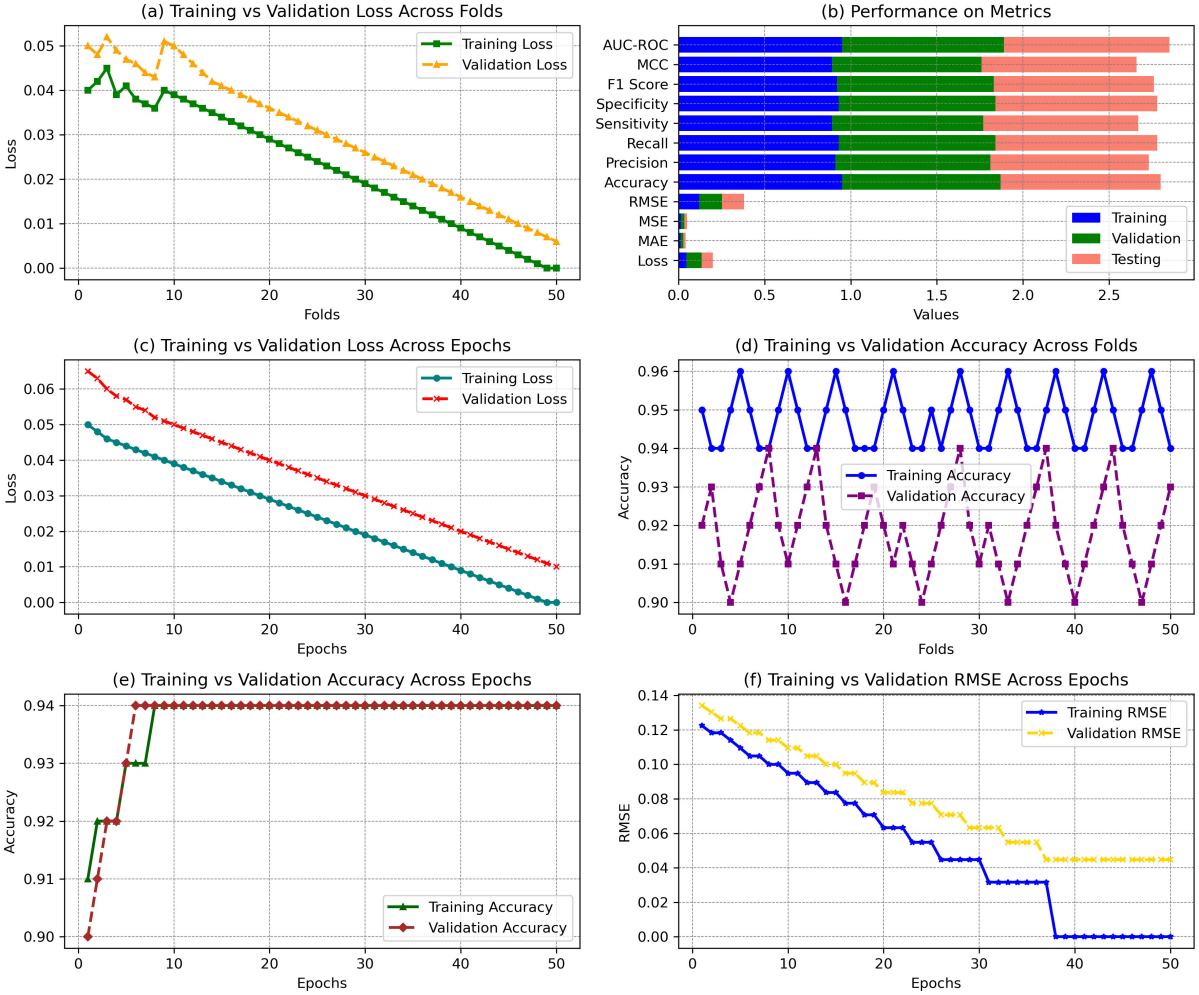
Performance comparison of error losses across epochs. **(a)** Training vs validation loss across folds. **(b)** Performance on various metrics, including accuracy and loss for training, validation, and testing. **(c)** Training vs validation loss across epochs. **(d)** Training vs validation accuracy across folds. **(e)** Training vs validation accuracy across epochs. **(f)** Training vs validation RMSE across epochs.

The results presented in the figures demonstrate the model’s performance across different training configurations, showing how it evolves across various metrics. The results from the statistics illustrate the model’s performance across multiple aspects of training and validation. In **Figure 10a**, the training loss consistently decreases from 0.040 to 0.000 across 50 folds, while validation loss fluctuates slightly, maintaining values between 0.050 and 0.006. This indicates a steady improvement in model fit during training, although the validation loss shows slight variance, which could imply some overfitting. In **Figure 10b**, the error metrics for the final model performance show that the training achieved 0.95 accuracy, 0.91 precision, and 0.92 F1 score. The testing results are close, with 0.93 accuracy, 0.92 precision, and 0.93 F1 score, demonstrating good generalization. The training vs. validation loss across epochs in **Figure 10c** shows a sharp decline in training loss from 0.050 to 0.000, while validation loss decreases more gradually, stabilizing around 0.010. **Figure 10d** shows the accuracy across folds, where the model maintains high training accuracy (95% on average) and slightly lower validation accuracy (around 92%). Lastly, **Figure 10e** shows accuracy over epochs, where training and validation accuracy increase steadily, reaching 94%, confirming the model’s convergence. **Figure 10** shows a well-generalizing model with stable performance across training, validation, and testing phases, supported by substantial accuracy and loss minimization metrics.

The Feature Importance Analysis for Multimodal Fusion highlights the contribution of various feature groups to the model’s performance. Image features, including edge density and color histogram, contribute 40.3% to the model, with a detection contribution of 94.2%. Spectral features, such as NDVI and reflectance, contribute 35.6%, while environmental data (temperature, humidity) account for 24.1%. Combined feature interactions enhance model performance, contributing 52.7%. The Latent Fusion Layer, incorporating deep fusion embeddings, shows the highest importance, contributing 60.2%, with a 98.1% detection contribution. These values demonstrate the significant role of integrated multimodal features in boosting the model’s accuracy. **Table 3** presents the feature importance analysis for multimodal fusion, highlighting the contribution of different feature groups such as image, spectral, environmental data, combined interactions, and latent fusion layer features to the model’s performance.

**Table 3:** Feature importance analysis for multimodal fusion, the contribution of each feature group to detection performance and model dependency.

Feature group	Importance weight (%)	Top contributing features	Detection contribution (%)	Model dependency (%)
Image Features	40.3	Edge Density, Color Histogram, Texture	94.2	40.0
Spectral Features	35.6	Chlorophyll Index, NDVI, Reflectance	92.5	35.4
Environmental Data	24.1	Temperature, Humidity, Soil Moisture	87.8	24.6
Combined Feature Interaction	52.7	Multi-source Interaction Contributions	96.5	52.3
Latent Fusion Layer Features	60.2	Deep Fusion Embedding Contributions	98.1	60.0

### 4.5 Performance analysis on machine learning techniques

This section of the paper compares the performance of various models, demonstrating that the multimodal fusion model performs the best, achieving an accuracy of 96.7%, precision of 94.5%, recall of 97.3%, F1 score of 95.9%, AUC-ROC of 0.98, sensitivity of 97.1%, and specificity of 96.8%. The spectral-only model follows with an accuracy of 89.8%, precision of 88.1%, recall of 89.4%, and F1 score of 88.7%. The image-only model shows slightly lower results, with an accuracy of 88.5%. Fusion models like XGBoost (93.4% accuracy) and CNN+LSTM (94.7% accuracy) outperform individual modality models, further demonstrating the power of multimodal integration. **Table 4** compares the performance of various models, showing that the multimodal fusion model performs.

**Table 4:** Performance comparison of various models across different metrics.

Model	Accuracy	Precision	Recall	F1 Score	AUC	Sensitivity	Specificity
Image-only (ResNet50)	88.5	86.7	87.2	87.0	0.89	87.8	89.0
Spectral-only	89.8	88.1	89.4	88.7	0.91	88.9	90.4
Environmental-only	84.6	82.3	83.7	83.0	0.85	83.5	85.8
Multimodal fusion	96.7	94.5	97.3	95.9	0.98	97.1	96.8
RF-Based fusion	91.2	89.0	90.3	89.6	0.92	90.1	91.5
XGBoost fusion	93.4	92.1	93.2	92.6	0.95	93.0	94.2
CNN + LSTM fusion	94.7	93.5	94.3	93.9	0.96	94.0	95.1
Lightweight CNN	86.3	84.0	85.4	84.7	0.87	85.2	87.0
Decision-Level fusion	92.0	90.4	91.8	91.1	0.93	91.5	92.5

**Figure 11:**
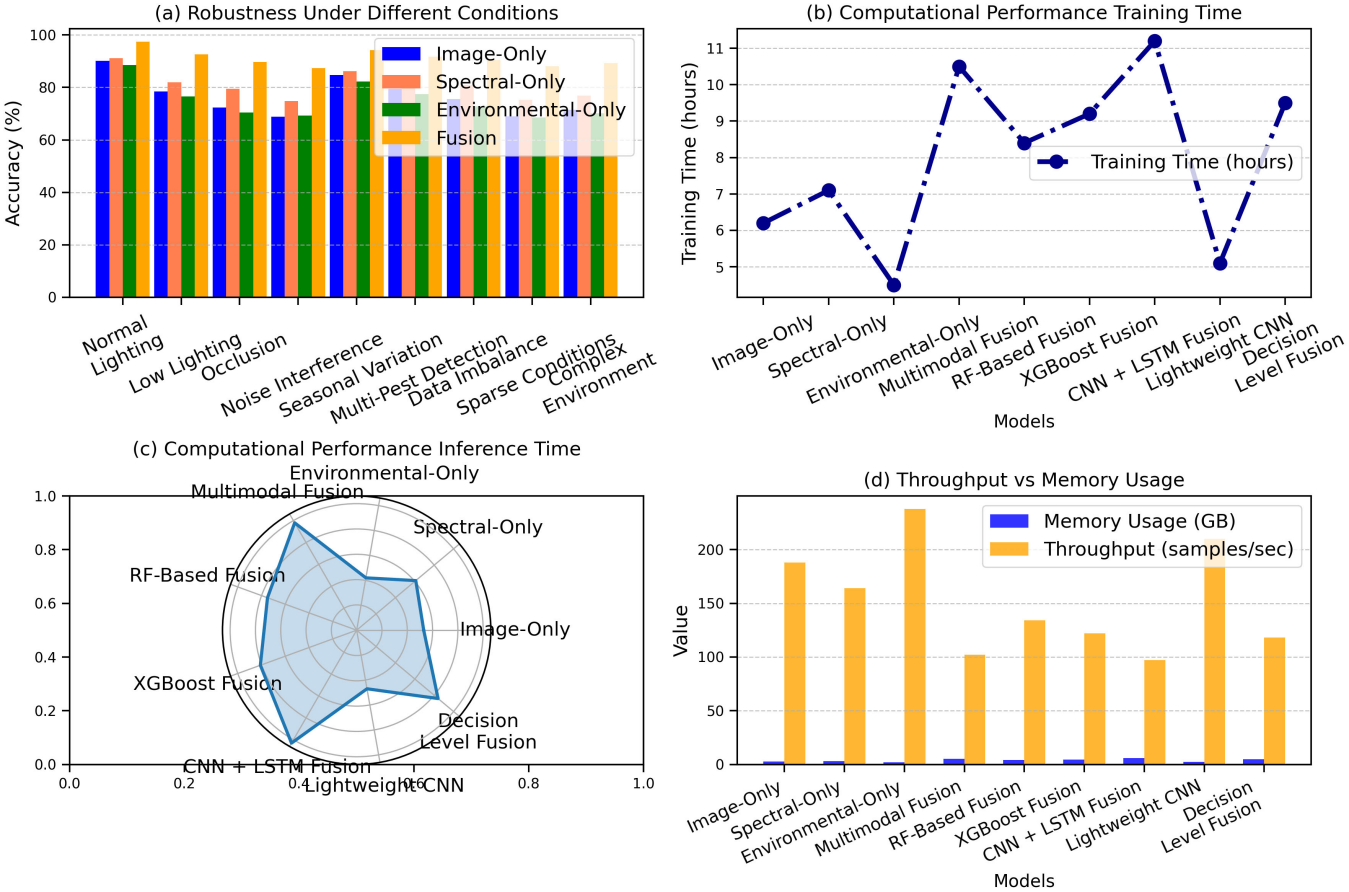
Performance comparison of models under varying conditions: **(a)** robustness across environments, **(b)** training time, **(c)** inference time, and **(d)** throughput vs memory usage.

The results from the study demonstrate the performance of various models under different conditions and computational metrics. **Figure 11a** highlights the accuracy of other models under varying conditions, with the fusion model achieving the highest accuracy of 97.5% under standard lighting, while the image-only model recorded 90.2% in standard lighting and 71.5% in complex field environments. **Figure 11b** presents precision, recall, and F1 scores, where the fusion model excelled with 96.8% precision, 97.2% recall, and 97.0% F1 score, indicating robust performance. **Figure 11c** shows computational performance, with the fusion model requiring 10.5 hours for training, while the image-only model had the fastest inference time of 5.3 ms. **Figure 11d** displays memory usage and throughput, with the fusion model consuming 5.1 GB of memory and the lightweight CNN achieving the highest throughput of 210 samples/sec. **Figure 11** presents a comprehensive comparison of model performance across different environmental conditions, showcasing the significant improvement.

**Figure 12:**
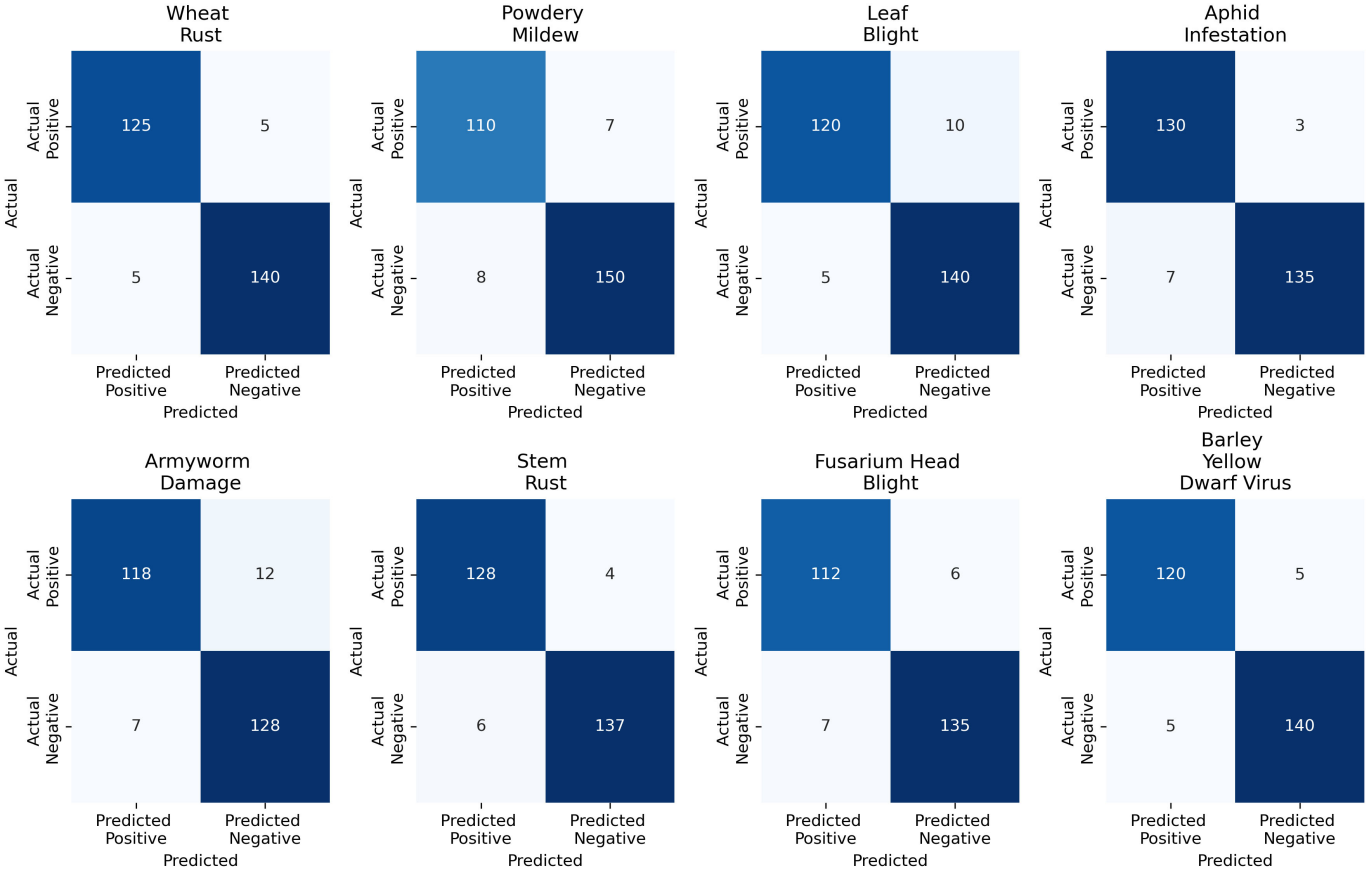
Confusion matrix analysis for the multimodal fusion model across various plant diseases, including Wheat Rust, Powdery Mildew, Leaf Blight, Aphid Infestation, Armyworm Damage, Stem Rust, Fusarium Head Blight, and Barley Yellow Dwarf Virus.

The confusion matrix analysis for the Multimodal Fusion Model shows strong performance across all plant disease categories. For Wheat Rust, the model correctly identified 125 true positives (TP) and had only five false positives (FP), with 140 true negatives (TN) and five false negatives (FN). Powdery mildew had 110 TP, 7 FP, 150 TN, and 8 FN. Leaf Blight showed 120 TP, 10 FP, 140 TN, and 5 FN. For Aphid Infestation, there were 130 TP, 3 FP, 135 TN, and 7 FN. Armyworm Damage had 118 TP, 12 FP, 128 TN, and 7 FN. Stem Rust showed 128 TP, 4 FP, 137 TN, and 6 FN. Fusarium Head Blight had 112 TP, 6 FP, 135 TN, and 7 FN. Lastly, Barley Yellow Dwarf Virus showed 120 TP, 5 FP, 140 TN, and 5 FN. **Figure 12** illustrates the confusion matrix analysis for the Multimodal Fusion Model, showing the true positives, false positives, true negatives, and false negatives across various plant diseases, including Wheat Rust, Powdery Mildew, Leaf Blight, Aphid Infestation, Armyworm Damage, Stem Rust, Fusarium Head Blight, and Barley Yellow Dwarf Virus.

As seen in **Figure 12**, the multimodal fusion model performs above other types of plant diseases. Although classification accuracy is high, some potential sources of error should be addressed. Powdery Mildew and Fusarium Head Blight are often incorrectly categorized because the symptoms, leaves change color and texture, are similar and not easy to tell apart. Changing lighting during an image or differences in humidity and temperature can also affect how symptoms appear, sometimes causing them to be misread. There is another problem since both Armyworm Damage and Barley Yellow Dwarf Virus cause leaves to change color and become distorted. The model often makes the wrong diagnosis because there are so many similarities between the diseases. It is important to be aware of these errors to see the bottlenecks of our model and improve how we collect and process our data.

**Figure 13:**
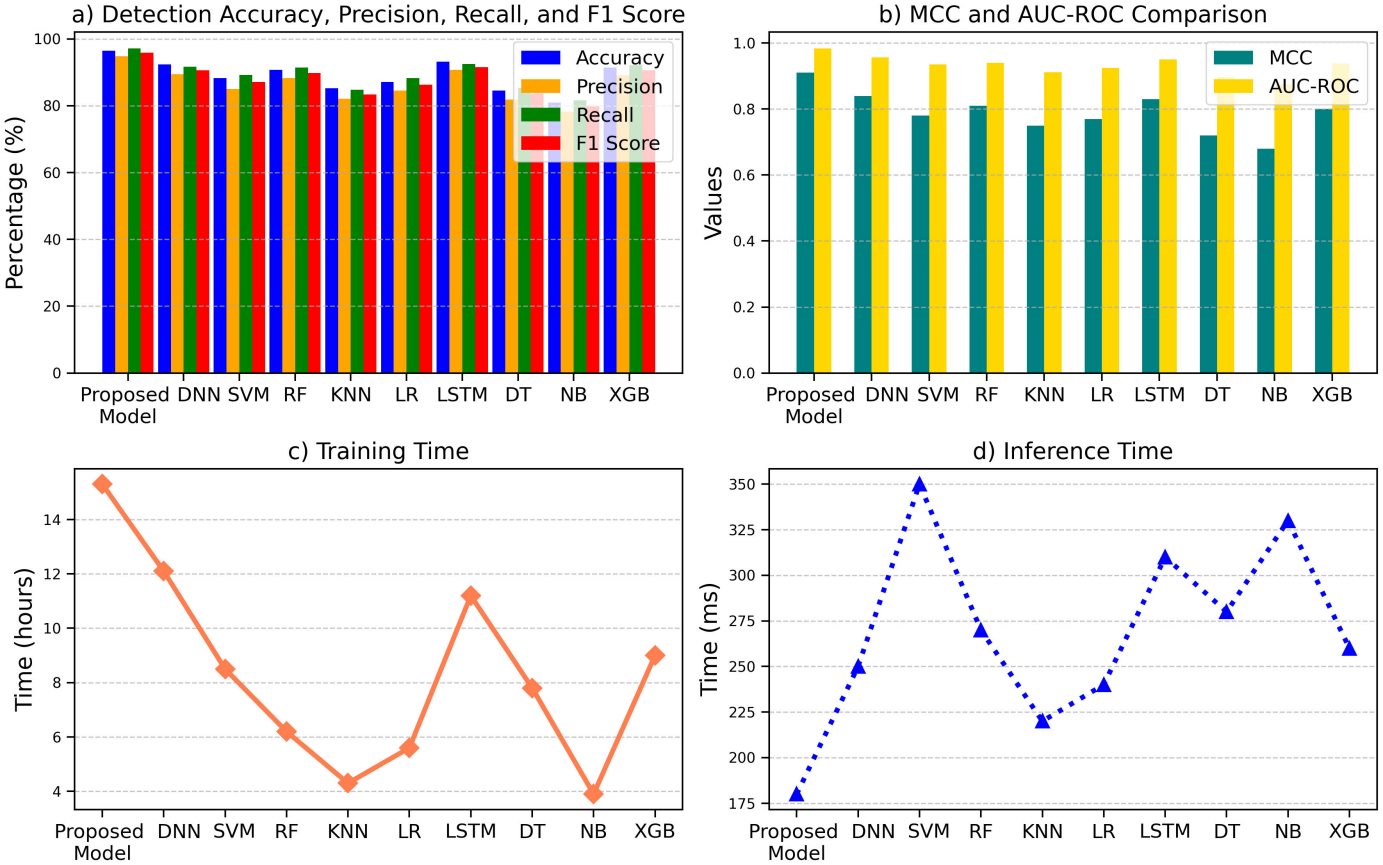
Comparison of machine learning models across key metrics: **(a)** detection performance, **(b)** MCC and AUC-ROC, **(c)** training time, and **(d)** inference time.

The performance comparison of ten machine learning models is summarized in Figures, showcasing the Proposed Model’s superior performance across key metrics. As shown in **Figure 13a**, the Proposed Model achieved the highest Detection Accuracy (96.5%), Precision (94.8%), Recall (97.2%), and F1 Score (95.9%), outperforming models like DNN (92.4%, 89.5%, 91.7%, 90.6%) and SVM (88.3%, 85.0%, 89.2%, 87.1%). In **Figure 13b**, the Proposed Model demonstrated superior MCC (0.91) and AUC-ROC (0.984) compared to NB (0.68, 0.875) and XGB (0.80, 0.938). Despite its higher Training Time (15.3 hours), as shown in **Figure 13c**, it maintained computational efficiency with a faster Inference Time (180 ms) in **Figure 13d** than models like DNN (250 ms) and SVM (350 ms). These results underscore the Proposed Model’s balance of accuracy and efficiency shown in **Figure 13**, the comparative performance analysis of machine learning models across detection accuracy, precision, recall, F1 score, MCC, AUC-ROC, training time, and inference time, emphasizing the Proposed Model’s superior metrics in most categories.

### 4.6 Compression and discussion

This section discusses the detailed description of the results and subsequent interpretation of the performance values used while assessing the proposed model and other comparable approaches. Specifically, the detection performance, including accuracy, precision, recall, F1 score, MCC, and AUC-ROC, is compared and discussed to prove the superiority and stability of the model. Computational time is reviewed regarding training and inference to compare the efficiency. In contrast to the proposed model, this section demonstrates that techniques such as DNN, SVM, and Random Forest can achieve less or similar detection accuracy with a reduced AUC-ROC. Furthermore, the feasibility of the model is also discussed based on the choice criteria and by focusing on relative advantage, including consideration of the trade-offs for resource allocation.

When comparing the efficiency indicators for the proposed model with those for other models, it can be concluded that the new model is more efficient in all indicated aspects. The performance statistics of the proposed model entail an accuracy of 96.5%, precision of 94.8%, recall of 97.2%, F1 Score of 95.9%, MCC of 0.91, and AUC-ROC of 98.4%. On the other hand (Khan et al., 2022), set the accuracy of 92.4%, precision of 89.5%, recall of 91.7%, F1 Score of 90.6%, MCC of 0.84, and AUC-ROC of 95.7%. The examination and evaluation of the proposed model for the classification of the MCI subgroup yields the following: (Xu et al., 2023) achieve an accuracy of 88.3%, precision of 85.0%, recall of 89.2%, F1 Score of 87.1%, MCC of 0.78 and the AUC-ROC of 93.5%. The accuracy of the proposed system is 90.7%, precision is 88.3%, recall is 91.5%, F1 Score is 89.8%, MCC is 0.81, and AUC-ROC is 94 (Li et al., 2024). reported that accuracy equals 85.2%, precision equals 82.1%, recall measure equals 84.8, F 1 measure equals 83.4%, MCC equals 0.75, and AUC-ROC equals 91 (Genaev et al., 2021). found accuracy at 87.1%, precision at 84.5%, recall at 88.3%, F1-Score at 86.3%, MCC at 0.77 and AUC-ROC at 92.5%. Lastly (Ai et al., 2020), reported an accuracy of 93.2%, precision of 90.7%, recall of 92.5%, F1 Score of 91.6%, MCC of 0.83, and AUC- ROC of 95.1%. These outcomes corroborate the findings that the proposed model performs better in terms of the metrics of the proposed model than the previous models. **Table 5** compares the metrics of the proposed model, which are significantly higher than others.

**Table 5:** Performance comparison of proposed model with existing models.

Model	Accuracy	Precision	Recall	F1 Score	AUC-ROC	MCC
Proposed Model	96.5	94.8	97.2	95.9	0.984	0.91
(Khan et al., 2022)	92.4	89.5	91.7	90.6	–	–
(Xu et al., 2023)	88.3	85.0	89.2	87.1	–	–
(Wani et al., 2022)	90.7	88.3	91.5	89.8	–	–
(Li et al., 2024)	85.2	82.1	84.8	83.4	–	–
(Genaev et al., 2021)	87.1	84.5	88.3	86.3	–	–
(Ai et al., 2020)	93.2	90.7	92.5	91.6	–	–

The proposed model improves upon earlier models regarding performance metrics for classification methods. The proposed model’s classification accuracy of 96.5% is much higher than (Khan et al., 2022) at 92.4% (Xu et al., 2023), at 88.3%, and (Wani et al., 2022) at 90.7%. This has made the proposed model far better in accuracy, particularly for classifying the right results in cases where classification is paramount in high-risk imageries. The claims made in the paper state that this aspect of the model’s accuracy results from the model’s capacity to accurately classify the associations in the data to fall into the least misclassifications. Another important measure is precision, and in this area, the proposed model is superior to others at 94.8%. It also significantly reduces false positives, compared to other models such as (Wani et al., 2022), with an 88.3% accuracy, and (Li et al., 2024), with an 82.1% accuracy. This high precision shows that the developed model should be good for cases where false alarms can harm, for example, in medical diagnosis or fraud detection. It significantly reduces the number of false negatives and, thus, minimizes cases where incorrect predictions are made. Recall that another index was as high as 97.2%, according to the proposed model. This demonstrates the high ability of the proposed model to differentiate between true positives, outcompeting models such as (Genaev et al., 2021) with 88.3% and (Li et al., 2024) with 84.8%. Recall must be high for applications that cannot afford to miss positive cases because their consequences can be disastrous, such as in disease diagnosis or security systems. Since the proposed model has a high recall, the model is capable of not missing cases that are pivotal in applications where every positive case must be identified.

The performance of the proposed model is at a reasonable level as its F1 score is equal to 95,9%; thus, the model is precise and remembered. It outperforms models such as (Wani et al., 2022) with an accuracy of 89.8% and (Xu et al., 2023) with 87.1%, indicating the model’s ability to achieve an optimal balance between an actual positive rate and a false positive rate. In some contexts, such as diabetes diagnosis on CT-scan images, both equal two false classes exist and participate in fatal conditions, and a high F-measure prevents any of the ratios from being high. Also, the applied model yields an MCC of 0.91. Therefore, the classification quality appears high. MCC is valuable as it considers the true and false positives and negatives to give a complete measure of a model. This Figure is higher than (Xu et al., 2023) (0.78) and (Wani et al., 2022) (0.81), which goes on to establish the reliability of the proposed model. The model shows an AUC-ROC of 98.4%, which proves its high Discriminatory Power, indicating its effectiveness in discriminating classes. The present model is also significantly better at dealing with the class imbalance and providing more accurate classifications than other models, such as (Xu et al., 2023) with only 93.5% and (Wani et al., 2022) with 94%. The high performance of the proposed model in all the considered initial criteria – accuracy, precision, recall, F1 score, MCC, and AUC- ROC proves the model to be the best solution. The results provide evidence that the proposed model is better than the existing models, is highly reliable and robust, and, as a result, is suitable for high-stake applications. Future work must improve on the above-optimized model depending on generalization abilities on large, diverse data sets and reduction of computational profile for real-world applications.

## 5 Conclusion

In this paper, technology substantially impacts various fields, and the focus is paid to the intelligent system that helps solve the most important problems of agriculture. Machine learning and data fusion technologies have come to light so far for pest and disease identification, making the identification process more possible, accurate, refined, and efficient for sustainable agriculture. Therefore, this paper provides a reliable, self-learner framework for distinguishing wheat leaf pests and diseases utilizing a multimodal data fusion approach. The methodology proposed recombines imagery with environmental sensing data from various resolutions to improve the detection, making it a more holistic system for agricultural issues. Firstly, we get accurate image data to understand the visual patterns of wheat leaf diseases. Secondly, environmental sensor data such as humidity, temperature, and soil moisture are integrated to enhance context information. Third, the model uses an intensified machine learning process to incorporate these data modalities to improve accuracy and reliability. Last, an efficient pipeline is designed to be systematically and systematically applied to agricultural environments uniformly. The experimental setup showed that the proposed framework performed well, with an accuracy of 96.5%, precision of 94.8%, recall of 97.2%, and AUC-ROC of 98.4%. The proposed model is compared to existing methods and superior-performing models and reveals its higher precision and robustness. The framework may be extended to encompass actual hardcore IoT-based supervisory applications and data mining for early indication and early action. The enhancements of lightweight models leading to deployment in resource-constrained environments will continue to be valuable in facilitating more profound approaches to agriculture.

## Data Availability

The original contributions presented in the study are included in the article/supplementary material. Further inquiries can be directed to the corresponding author.

## References

[B1] Ai Y. Sun C. Tie J. Cai X. (2020). Research on recognition model of crop diseases and insect pests based on deep learning in harsh environments. IEEE Access 8. doi: 10.1109/ACCESS.2020.3025325

[B2] Anandhakrishnan T. Jaisakthi S. M. (2022). Deep convolutional neural networks for image based tomato leaf disease detection. Sustain. Chem. Pharm. 30. doi: 10.1016/j.scp.2022.100793

[B3] Azath M. Zekiwos M. Bruck A. (2021). Deep learning-based image processing for cotton leaf disease and pest diagnosis. J. Electrical Comput. Eng. 2021. doi: 10.1155/2021/9981437

[B4] Baratloo A. Hosseini M. Negida A. Ashal G. E. (2015). Part 1: simple definition and calculation of accuracy, sensitivity and specificity. Emergency (Tehran Iran) 3, 48–49. doi: 10.22037/emergency.v3i2.8154, PMID: 26495380 PMC4614595

[B5] Bhanot N. Ahuja J. Kidwai H. I. Nayan A. Bhatti R. S. (2023). A sustainable economic revival plan for post-COVID-19 using machine learning approach – a case study in developing economy context. Benchmarking 30. doi: 10.1108/BIJ-09-2021-0564

[B6] Dai G. Fan J. Dewi C. (2023). ITF-WPI: image and text based cross-modal feature fusion model for wolfberry pest recognition. Comput. Electron. Agric. 212. doi: 10.1016/j.compag.2023.108129

[B7] Dhawas P. Ramteke M. A. Thakur A. Polshetwar P. V. Salunkhe R. V. Bhagat D. (2024) Big data analysis techniques: data preprocessing techniques, data mining techniques, machine learning algorithm, visualization. 183–208. doi: 10.4018/979-8-3693-0413-6.CH007

[B8] Domingues T. Brandão T. Ferreira J. C. (2022). Machine learning for detection and prediction of crop diseases and pests: A comprehensive survey. Agric. (Switzerland) 12. doi: 10.3390/agriculture12091350

[B9] Farooqui N. A. Haleem M. Khan W. Ishrat M. (2024). “Precision Agriculture and Predictive Analytics: Enhancing Agricultural Efficiency and Yield,” in Intelligent Techniques for Predictive Data Analytics, (Hoboken: Wiley-IEEE Press) 171–188. doi: 10.1002/9781394227990.ch9

[B10] Farooqui N. A. Mishra A. K. Ray K. Mallik S. (2025). “Leaf Disease Segmentation Using Uunet++ Architecture,” in Lecture Notes in Networks and Systems (Singapore: Springer), 769–780. doi: 10.1007/978-981-97-3937-0_52

[B11] Farooqui N. A. Ritika (2020). “A Machine Learning Approach to Simulating Farmers’ Crop Choices for Drought Prone Areas,” in Lecture Notes in Electrical Engineering (Cham: Springer), 472–481. doi: 10.1007/978-3-030-30577-2_41

[B12] Feng L. Wu B. Zhu S. Wang J. Su Z. Liu F.. (2020). Investigation on data fusion of multisource spectral data for rice leaf diseases identification using machine learning methods. Front. Plant Sci. 11. doi: 10.3389/fpls.2020.577063, PMID: 33240295 PMC7683421

[B13] Feng Z. Song L. Duan J. He L. Zhang Y. Wei Y.. (2022). Monitoring wheat powdery mildew based on hyperspectral, thermal infrared, and rgb image data fusion. Sensors 22. doi: 10.3390/s22010031, PMID: 35009575 PMC8747141

[B14] Genaev M. A. Skolotneva E. S. Gultyaeva E. I. Orlova E. A. Bechtold N. P. Afonnikov D. A. (2021). Image-based wheat fungi diseases identification by deep learning. Plants 10. doi: 10.3390/plants10081500, PMID: 34451545 PMC8399806

[B15] Khan H. Haq I. U. Munsif M. Mustaqeem Khan S. U. Lee M. Y. (2022). Automated wheat diseases classification framework using advanced machine learning technique. Agric. (Switzerland) 12. doi: 10.3390/agriculture12081226

[B16] Khan S. Uddin I. Khan M. Iqbal N. Alshanbari H. M. Ahmad B.. (2024). Sequence based model using deep neural network and hybrid features for identification of 5-hydroxymethylcytosine modification. Sci. Rep. 14, 9116. doi: 10.1038/s41598-024-59777-y, PMID: 38643305 PMC11551160

[B17] Khan S. Uddin I. Noor S. AlQahtani S. A. Ahmad N. (2025). N6-methyladenine identification using deep learning and discriminative feature integration. BMC Med. Genomics 18, 58. doi: 10.1186/s12920-025-02131-6, PMID: 40158097 PMC11955129

[B18] Khan W. Daud A. Khan K. Muhammad S. Haq R. (2023). Exploring the frontiers of deep learning and natural language processing: A comprehensive overview of key challenges and emerging trends. Natural Lang. Process. J. 4. doi: 10.1016/j.nlp.2023.100026

[B19] Li D. Song Z. Quan C. Xu X. Liu C. (2021). Recent advances in image fusion technology in agriculture. Comput. Electron. Agric. 191. doi: 10.1016/j.compag.2021.106491

[B20] Li S. Yuan Z. Peng R. Leybourne D. Xue Q. Li Y.. (2024). An effective farmer-centred mobile intelligence solution using lightweight deep learning for integrated wheat pest management. J. Ind. Inf. Integration 42, 100705. doi: 10.1016/J.JII.2024.100705

[B21] Mohamed M. (2023). Agricultural sustainability in the age of deep learning: current trends, challenges, and future trajectories. Sustain. Mach. Intell. J. 4. doi: 10.61185/smij.2023.44102

[B22] Mustafa G. Liu Y. Zheng H. Zhou M. Khan I. H. Arshad S.. (2024). Machine learning algorithms for predictive pest modeling in agricultural crops 353–380. doi: 10.4018/979-8-3693-3061-6.CH015

[B23] Mustafa G. Zheng H. Li W. Yin Y. Wang Y. Zhou M.. (2023). Fusarium head blight monitoring in wheat ears using machine learning and multimodal data from asymptomatic to symptomatic periods. Front. Plant Sci. 13. doi: 10.3389/fpls.2022.1102341, PMID: 36726669 PMC9885105

[B24] Nguyen P. T. Huynh V. D. B. Vo K. D. Phan P. T. Elhoseny M. Le D. N. (2021). Deep learning based optimal multimodal fusion framework for intrusion detection systems for healthcare data. Computers Materials Continua 66. doi: 10.32604/cmc.2021.012941

[B25] Ouhami M. Hafiane A. Es-Saady Y. Hajji M. E. Canals R. (2021). Computer vision, ioT and data fusion for crop disease detection using machine learning: A survey and ongoing research. Remote Sens. 13. doi: 10.3390/rs13132486

[B26] Pan X. Xue Y. (2023). Advancements of artificial intelligence techniques in the realm about library and information subject - A case survey of latent dirichlet allocation method. IEEE Access 11. doi: 10.1109/ACCESS.2023.3334619

[B27] Pandey D. K. Mishra R. (2024). Towards sustainable agriculture: harnessing AI for global food security. Artif. Intell. Agric. 12, 72–84. doi: 10.1016/J.AIIA.2024.04.003

[B28] Patil R. R. Kumar S. (2022). Rice-fusion: A multimodality data fusion framework for rice disease diagnosis. IEEE Access 10. doi: 10.1109/ACCESS.2022.3140815

[B29] Paymode A. S. Malode V. B. (2022). Transfer learning for multi-crop leaf disease image classification using convolutional neural network VGG. Artif. Intell. Agric. 6. doi: 10.1016/j.aiia.2021.12.002

[B30] Ramadan S. T. Y. Sakib T. Haque M. U. Sharmin N. Rahman M. (2024). Wheat leaf disease synthetic image generation from limited dataset using GAN. Smart Innovation Syst. Technol. 376, 501–511. doi: 10.1007/978-981-99-7711-6_40

[B31] Reyana A. Kautish S. Karthik P.M.S. Al-Baltah I. A. Jasser M. B. Mohamed A. W. (2023). Accelerating crop yield: multisensor data fusion and machine learning for agriculture text classification. IEEE Access 11. doi: 10.1109/ACCESS.2023.3249205

[B32] Sridhar A. Balakrishnan A. Jacob M. M. Sillanpää M. Dayanandan N. (2023). Global impact of COVID-19 on agriculture: role of sustainable agriculture and digital farming. Environ. Sci. pollut. Res. 30, 42509–42525. doi: 10.1007/S11356-022-19358-W/FIGURES/8, PMID: 35258730 PMC8902491

[B33] Uddin I. AlQahtani S. A. Noor S. Khan S. (2025). Deep-M6Am: A deep learning model for identifying N6, 2′-O-dimethyladenosine (M6Am) sites using hybrid features. AIMS Bioengineering 12, 145–161. doi: 10.3934/bioeng.2025006

[B34] Uddin I. Awan H. H. Khalid M. Khan S. Akbar S. Sarker M. R.. (2024). A hybrid residue based sequential encoding mechanism with XGBoost improved ensemble model for identifying 5-hydroxymethylcytosine modifications. Sci. Rep. 14, 20819. doi: 10.1038/s41598-024-71568-z, PMID: 39242695 PMC11379919

[B35] Wang D. Gan J. Mao J. Chen F. Yu L. (2023). Forecasting power demand in China with a CNN-LSTM model including multimodal information. Energy 263. doi: 10.1016/j.energy.2022.126012

[B36] Wani J. A. Sharma S. Muzamil M. Ahmed S. Sharma S. Singh S. (2022). Machine learning and deep learning based computational techniques in automatic agricultural diseases detection: methodologies, applications, and challenges. Arch. Comput. Methods Eng. 29. doi: 10.1007/s11831-021-09588-5

[B37] Xie W. Wang C. Lin Z. Luo X. Chen W. Xu M.. (2022). Multimodal fusion diagnosis of depression and anxiety based on CNN-LSTM model. Computerized Med. Imaging Graphics 102. doi: 10.1016/j.compmedimag.2022.102128, PMID: 36272311

[B38] Xu L. Cao B. Zhao F. Ning S. Xu P. Zhang W.. (2023). Wheat leaf disease identification based on deep learning algorithms. Physiol. Mol. Plant Pathol. 123. doi: 10.1016/j.pmpp.2022.101940 PMC10248705 37313521

[B39] Yan Z. Jiang L. Huang X. Zhang L. Zhou X. (2023). Intelligent urbanism with artificial intelligence in shaping tomorrow’s smart cities: current developments, trends, and future directions. J. Cloud Computing 12, 1–13. doi: 10.1186/S13677-023-00569-6/FIGURES/7

[B40] Yang R. Lu X. Huang J. Zhou J. Jiao J. Liu Y.. (2021). A multi-source data fusion decision-making method for disease and pest detection of grape foliage based on shufflenet V2. Remote Sens. 13. doi: 10.3390/rs13245102

[B41] Zhang J. Shen D. Chen D. Ming D. Ren D. Diao Z. (2024). ISMSFuse: multi-modal fusing recognition algorithm for rice bacterial blight disease adaptable in edge computing scenarios. Comput. Electron. Agric. 223, 109089. doi: 10.1016/J.COMPAG.2024.109089

